# Ferrocene derivatives with planar chirality and their enantioseparation by liquid‐phase techniques

**DOI:** 10.1002/elps.202200148

**Published:** 2022-09-04

**Authors:** Paola Peluso, Victor Mamane

**Affiliations:** ^1^ Istituto di Chimica Biomolecolare ICB CNR Sede secondaria di Sassari Sassari Italy; ^2^ Institut de Chimie de Strasbourg, UMR 7177 CNRS‐Université de Strasbourg Strasbourg France

**Keywords:** enantioseparation, ferrocenes, metallocenes, planar chirality, polysaccharide‐based chiral stationary phases

## Abstract

In the last decade, planar chiral ferrocenes have attracted a growing interest in several fields, particularly in asymmetric catalysis, medicinal chemistry, chiroptical spectroscopy and electrochemistry. In this frame, the access to pure or enriched enantiomers of planar chiral ferrocenes has become essential, relying on the availability of efficient asymmetric synthesis procedures and enantioseparation methods. Despite this, in enantioseparation science, these metallocenes were not comprehensively explored, and very few systematic analytical studies were reported in this field so far. On the other hand, enantioselective high‐performance liquid chromatography has been frequently used by organic and organometallic chemists in order to measure the enantiomeric purity of planar chiral ferrocenes prepared by asymmetric synthesis. On these bases, this review aims to provide the reader with a comprehensive overview on the enantioseparation of planar chiral ferrocenes by discussing liquid‐phase enantioseparation methods developed over time, integrating this main topic with the most relevant aspects of ferrocene chemistry. Thus, the main structural features of ferrocenes and the methods to model this class of metallocenes will be briefly summarized. In addition, planar chiral ferrocenes of applicative interest as well as the limits of asymmetric synthesis for the preparation of some classes of planar chiral ferrocenes will also be discussed with the aim to orient analytical scientists towards ‘hot topics’ and issues which are still open for accessing enantiomers of ferrocenes featured by planar chirality.

**Abbreviations**
2‐PrOH: 2‐propanol
B3LYP: Becke–3‐parameter–Lee–Yang–Parr
B3PW91: Becke88, Perdew–Wang
CCSD: coupled cluster singles and doubles
CCSD(T): coupled cluster singles and doubles (triples)
CD: cyclodextrin
CIP: Cahn–Ingold–Prelog
Cp: cyclopentadienyl
CSP: chiral stationary phase
DEA: diethylamine
def2‐TZVP: default2‐valence triple‐zeta polarization
def2‐TZVPP: default2‐valence triple‐zeta with two sets of polarization functions
DFT: density functional theory
*ee*
: enantiomeric excess
EEO: enantiomer elution order
EtOH: ethanol
Fc: ferrocene
HB: hydrogen bond
IUPAC: International Union of Pure and Applied Chemistry
LANL2DZ: Los Alamos National Laboratory 2‐double‐z
MD: molecular dynamics
MeOH: methanol
MP: Møller–Plesset
PBE: Perdew–Burke–Ernzerhof
pinB: boron pinacolate
SCE: saturated calomel electrode
SFC: supercritical fluid chromatography
TEA: triethylamine
*V*
: electrostatic potential
*V*_S,max_
: electrostatic potential maximum
*V*_S,min_
: electrostatic potential minimum
XB: halogen bond

## 1 INTRODUCTION

The concept of planar chirality was introduced in the mid‐20th century [1] and subsequently discussed in a number of seminal papers focused on fundamentals of stereochemistry and stereoisomerism [2, 3, 4, 5]. Later, in the International Union of Pure and Applied Chemistry (IUPAC) recommendations on the *Basic terminology of stereochemistry* [6], ‘planar chirality’ was defined as the *Term used by some authorities to refer to stereoisomerism resulting from the arrangement of out‐of‐plane groups with respect to a plane (chirality plane)*. On this basis, in planar chiral compounds such as cyclophanes, rigid alkenes and metallocenes, chirality arises from the different arrangements of groups located at both sides of a planar core. This feature induces a breaking of symmetry generating a chiral plane as the element of chirality (stereogenic unit) defining the stereochemical properties of this type of chiral compounds [7].

**FIGURE 1:**
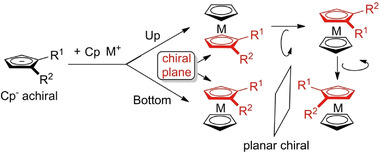
Planar chirality of metallocenes

Metallocenes are organometallic coordination compounds in which one atom of a transition metal (iron, ruthenium, manganese, osmium, titanium and zirconium) is bonded to the face of two cyclopentadienyl [η^5^‐(C_5_H_5_)] (Cp) ligands which lie in parallel planes [8]. In accord with the IUPAC recommendations on class names of organic compounds, the term ‘metallocene’ should not be used for analogues having rings other than Cp as ligand [8]. Planar chirality of metallocenes is related to their three‐dimensional sandwich‐like spatial arrangement where a transition metal is situated between two Cp ligands. Cp anions (Cp^−^) are achiral flat‐shaped chemical species which upon disubstitution (R_1_ ≠ R_2_) presents two enantiotopic faces. Thus, π‐coordination of the planar prochiral Cp^−^ to a CpM^+^ (metal cyclopentadienyl cation) discriminates the two enantiotopic faces to induce planar chirality in the metallocene complexes. In other words, disubstitution (in 1,2‐ or 1,3‐positions) of one of the Cp ligands of metallocenes generates a chiral plane (coloured in red, Figure 1) where the two sides of the plane are differently occupied.

The most prominent member of the metallocene family is ferrocene (M=Fe) (Fc) [9], the first sandwich complex discovered in early 1950s [10, 11] and structurally

elucidated shortly after [12, 13, 14]. Due to its high stability and similar reactivity to benzene, the ferrocene chemistry developed rapidly and until the late 1960s, a large number of planar chiral ferrocenes were reported, in particular by the group of Schlögl [15, 16].

**FIGURE 2:**
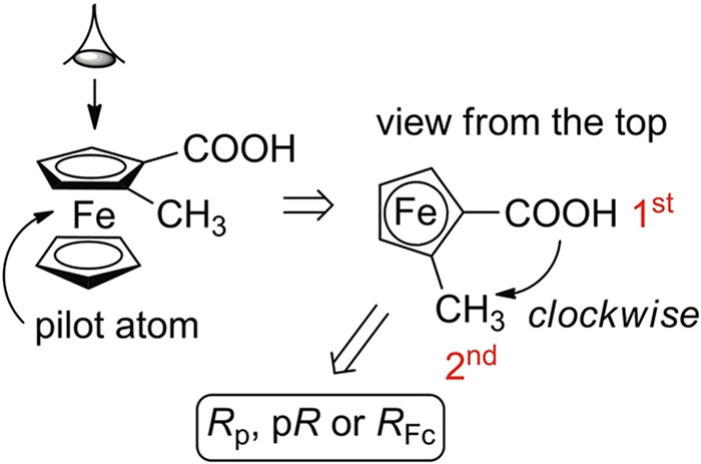
Absolute configuration of planar chiral ferrocenes

The nomenclature rule proposed by Schlögl [17] to define the absolute configuration of planar chiral ferrocene compounds proceeds in three steps. First, considering the chiral plane, iron is assigned as the pilot atom (Figure 2) that is the linked atom being out of the chiral plane and with the highest priority based on the Cahn–Ingold–Prelog (CIP) priority rules [18]. Second, the chiral plane is observed from the top, thus positioning the iron atom beneath the plane. Third, the two substituents on the Cp are classified according to CIP priority rules: if the relative orientation from the first‐priority substituent to the second one is clockwise, the molecule is assigned as an (*R*)‐stereoisomer; if it is counterclockwise, the molecule is assigned as an (*S*)‐stereoisomer (Figure 2). Because central and planar chirality may be both present in ferrocene derivatives, the planar chirality of ferrocene is usually differentiated by adding the letter ‘p’ (*R*_p_ and *S*_p_ or p*R* and p*S*) or the acronym ‘Fc’ (*R*_Fc_ or *S*_Fc_) to specify that the nomenclature corresponds to the planar chirality. Actually, there are other (*R*)/(*S*)‐nomenclature rules to define the absolute configurations in planar chiral iron complexes which possess π‐ligands [19]. However, the nomenclature proposed by Schlögl remains the most used in planar chiral ferrocene chemistry.

**FIGURE 3:**
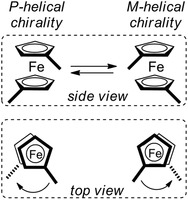
Side and top views of *P* and *M* helical chirality of the ferrocene moiety of 1,*n*′‐disubstituted ferrocenes

It is worth mentioning that, in principle, *P* and *M* helical enantiomeric conformations (*P* [plus] and *M* [minus], respectively, represent the right‐ and left‐handed helicities) are present in the 1,*n*′‐disubstituted ferrocenes (Figure 3). However, they are interconvertible based on a torsional twist about the Cp(centroid)–Fe–Cp(centroid) axis [20]. Thus, 1,*n*′‐disubstituted ferrocenes are not chiral unless electronic or steric constraints impede the torsional twist about the Cp–Fe–Cp axis.

Over time, the main field of application of planar chiral ferrocenes has been asymmetric catalysis. It was in the early 1990s that the usefulness of chiral ferrocenylphosphines in asymmetric catalysis was recognized [21, 22], and an impressive number of planar chiral ferrocenes were produced on the basis of diastereoselective methodologies implying the use of different chiral auxiliaries [23, 24, 25]. The majority of chiral ferrocene ligands available so far have both central and planar chirality [21]. In some cases, both chiral elements proved to act synergistically (matched combination), providing high asymmetric induction [26, 27]. On the other hand, there are a growing number of ferrocene ligands with only planar chirality that provide excellent enantioselectivities [28, 29]. In the last decade, planar chiral ferrocenes have been attracting a growing interest beyond asymmetric synthesis, finding application in other fields such as medicinal chemistry [30], chiroptical spectroscopy [31, 32, 33] and electrochemistry [34, 35].

**FIGURE 4:**
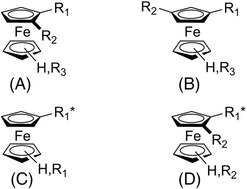
Chiral ferrocene motifs studied in enantioseparation science (R_1_* = chiral framework): planar chiral ferrocenes containing only a chiral plane as a stereogenic unit (A,B); chiral ferrocenes featured by a single chiral centre as a stereogenic unit (C); planar chiral ferrocenes featured by both central and planar chirality (D)

In 1970, Ugi et al. reported a pioneering work on the diastereoselective synthesis of 1,2‐disubstituted ferrocene derivatives starting from enantiopure *N*,*N*‐dimethyl‐1‐ferrocenylethylamine [36]. In addition, methodologies based on the direct enantioselective synthesis from prochiral ferrocenes have been developed [37, 38]. Liquid‐phase enantioseparation can also be used to access pure or enriched enantiomers of planar chiral ferrocenes [39]. A reason which makes the availability of efficient enantioseparation methods urgent is that, in some cases, asymmetric synthesis procedures for accessing enantiomerically enriched ferrocenes not always provide products with satisfactory enantiomeric purity [40, 41, 42]. Moreover, for a long time the preparation of pure enantiomers of certain planar chiral ferrocenes, for instance those containing a pyridine core, required diastereomeric resolution or enantioselective high‐performance liquid chromatography (HPLC) separation [38, 43]. However, despite the growing interest in planar chiral ferrocenes containing only a chiral plane as a stereogenic unit (Figure 4A, B), the enantioseparation of this type of compounds was not comprehensively explored, and very few systematic analytical studies were reported so far [44, 45, 46, 47, 48]. On the other hand, enantioselective HPLC has been frequently used in organic and organometallic chemistry studies in order to measure the enantiomeric purity of planar chiral ferrocenes prepared by asymmetric synthesis. In these cases, the chromatographic analyses have been in general performed under normal phase conditions, this trend being likely due to the fact that non‐analytical scientists are more familiar with this elution mode compared to polar organic and reversed‐phase modes. Over time, several methods were also developed for the enantioseparation of chiral ferrocenes featured by a single chiral centre as a stereogenic unit (Figure 4C) [49, 50, 51, 52, 53] or by both central and planar chirality (Figure 4D) [48].

Given this context and aiming to provide the reader with a comprehensive overview of the field, this review describes liquid‐phase enantioseparation of planar chiral ferrocenes, integrating this main topic with other relevant aspects of ferrocene chemistry. For this purpose, in the first part, introductive topics will be presented: (a) structural features of ferrocenes (Section 1.1); (b) the main computation tools currently available to model ferrocene structures will also be briefly summarized (Section 1.2). In this regard, it is worth mentioning that in modern enantioseparation science, integrating experimental and theoretical data has proven to be a useful tool to understand the enantioseparation mechanisms at molecular level. Approaches to model HPLC enantioseparation of planar chiral ferrocenes were not reported until very recently [45]; (c) applications which justify the growing interest in planar chiral ferrocene chemistry (Section 2.1); (d) the main asymmetric syntheses available to access enantiomerically enriched planar chiral ferrocenes, highlighting advances and limitations of the synthetic approaches (Section 2.2).

In the second part (Section 3), with the aim to profile the historical advancement of the field as well as the‐state‐of‐the‐art of liquid‐phase enantioseparations of planar chiral ferrocenes, methods, techniques and chiral stationary phases (CSPs) used in this field over time will be overviewed, highlighting the relationships between the structural features of the analytes and the chromatographic outcomes.

### 1.1 General features of the ferrocene structure

Besides the possibility of being chiral, ferrocene possesses exceptional structural and electronic characteristics [9]. With an iron centre that has the saturated electronic structure of krypton, ferrocene is thermally stable up to 400°C, tolerant to light, oxygen and moisture. It has good solubility in all common organic solvents and is easily functionalized by common procedures. The Cp rings in ferrocenes carry a partial negative charge; thus, they have electron donor properties and may undergo electrophilic substitution [54]. Owing to these features, over time ferrocene derivatives have found application in several fields such as catalysis [22], material sciences [55, 56, 57, 58] and medicinal chemistry [59, 60, 61]. Indeed, ferrocene presents low toxicity that allows its use as a bioisostere of aryl and heteroaryl groups [30, 62, 63]. Moreover, ferrocene possesses mild and reversible oxidation around +0.4 V versus saturated calomel electrode. Thus, the ferrocenium/ferrocene system (Fc^+^/Fc) is a versatile redox couple which was shown to be suitable for the preparation of switchable functional systems [64].

An interesting structural feature of ferrocene is that the inter‐ring distance between the two Cp rings is about 3.3 Å, which is suitable for hydrogen bonding (HB) [20], and in principle for other noncovalent interactions, between attached groups on the two Cp rings [65]. Recently, by integrating experimental and computational analyses, it was demonstrated that in chiral ferrocenes, featured by central and/or planar chirality, intra‐ and intermolecular HBs involving neighbouring groups may affect the conformational properties of the ferrocene system in terms of eclipsed (*D5h*) or staggered (*D5d*) structures of the two Cp rings (Figure S1), as well as their chiroptical responses [33, 66]. In this regard, the eclipsed conformation is reported to be the most stable one at room temperature for ferrocene in solution [67, 68], and for mono‐substituted ferrocenes [68, 69]. Density functional theory (DFT) computations showed that the interconversion barrier between equivalent eclipsed conformers (stable minima) and the staggered conformers (saddle points) is small in ferrocene [66, 70]. The *D5d*–*D5h* barrier to internal rotation of the ligand rings was estimated at 0.9 kcal/mol [71]. Otherwise, in some cases, the staggered conformation was shown to be favoured by intermolecular interactions in the crystalline state [72].

### 1.2 Basic concepts of ferrocene modelling

In the last few years, the integration of computational and experimental analysis has been developing as a pivotal tool to disclose enantioselective recognition mechanisms at molecular level [73, 74, 75]. In this field, the selection of the proper combination of method, functional and basis set for geometry optimization and calculations of molecular properties of the selected structures is essential in order to obtain reliable theoretical information. However, in general this choice is guided by the need to find the best compromise between calculation reliability and computational time required for performing the calculations with a given method. The coupled cluster singles and doubles (CCSD) and triples (CCSD(T)) methods are considered the ‘gold standard’ quantum chemical methods, but with a computational time cost which is not negligible. Early Hartree–Fock calculations for geometry optimization of ferrocene were performed in the 1980s by Lüthi et al. providing a metal–ligand distance of 1.88 Å [76], in poor agreement with the experimental value of 1.66 Å [71, 77]. In 1996, Koch, Jørgensen and Helgaker performed CCSD and CCSD(T) calculations on (staggered) ferrocene and obtained metal–ligand distances of 1.672 and 1.660 Å, respectively, in close agreement with the experimental data [78]. In 2006, Coriani et al. compared different functionals to model the equilibrium structure of ferrocene using the Møller–Plesset (MP2), CCSD and CCSD(T) models [70]. Although the MP2 model failed, the CCSD(T) results agreed very well with the experimental bond lengths. Moreover, the comparison with DFT results indicated that the Becke–Lee–Yang–Parr B3LYP model yields the best results in comparison to both experimental data and CCSD(T) calculations. In particular, B3LYP provided an error of about 0.02 Å for the metal–ligand distance and smaller errors for the cyclopentadienyl rings (up to 0.01 Å). Otherwise, the Becke–Perdew BP86 method afforded a metal–carbon distance only 0.001 Å off experimental values, but rather large deviations in the cyclopentadienyl C–C and C–H bonds. Full geometry optimization of ferrocenes was also performed by using the functional B3LYP and the m6‐31G(d) as basis set that incorporates necessary diffuse d‐type functions for the first‐row transition metals such as Fe, thus exhibiting better performances than the conventional 6–31G(d) basis set for the iron atom in ferrocene [79, 80]. Latouche et al. tested different combinations of functionals and basis sets for metallocenes including ferrocene [81]. Although the Perdew–Burke–Ernzerhof (PBE0) functional underestimated the Fe−Cp distance, the best agreement for this distance was obtained at the Coulomb‐attenuating method−B3LYP and B3PW91 levels. On this basis, recently Barone et al. used the hybrid functional B3PW91 with the def2TZVP as basis set for DFT geometry optimization and frequency calculations of substituted ferrocenes [32, 33, 66]. Yáñez‐S et al. reported the full geometry optimization of the ground state of the 2‐ferrocenyl‐1,8‐naphthyridine at the B3LYP/6‐31++G(d) level of theory, and the LANL2DZ basis set with the effective core potential was used to describe Fe [82]. The comparison of calculated and crystallographic structures showed that both geometries (eclipsed) were nearly equal with slight deviation in the cyclopentadienyl rings and in the naphthyridine region [82]. A similar computational method (B3LYP/LANL2DZ(Fe,I)/6‐31G(d)) was recently used by Erb et al. for geometry optimization and calculations of molecular properties of a series of *N*,*N*‑dialkylferrocenesulfonamides [83].

In 2011, Bogdanović and Novaković evaluated the frequency of occurrence of the ferrocene dimer in crystals reported in the Cambridge Structural Databank [84], finding that 46.8% of ferrocene derivative crystals contained a dimer. Vargas‐Caamal et al. showed by computations that dispersion is the major contribution to stabilize a metallocene dimer [85]. In this study, final equilibrium geometries were calculated at PBE/def2‐TZVP and PBE‐D2/def2‐TZVP levels, the latter approach including the D2 version of Grimme's dispersion corrections [86]. Indeed, DFT methods have shown fundamental problems to describe properly dispersion forces that require suitable methods or corrected DFT methods for a proper description. On the other hand, the impact of dispersion on geometry optimization and calculation of some properties of molecules in their unbound state may be low. Recently, our groups optimized the geometry of halogenated ferrocenes **1**–**5** (Table S1), in their unperturbed isolated state in the vacuum, at DFT level of theory, by using the B3LYP functional with [87] and without [46] D3 dispersion correction, and the Ahlrichs‐type triple‐ζ valence def2‐TZVPP basis set. Under these conditions, the local maxima of the electrostatic potential (*V*_S,max_) values were calculated on the 0.002 au isodensity surface. As expected, dispersion corrections provided less positive *V*_S,max_ values and smaller variations as the structure of the ferrocene changes. However, the variation of the relative magnitudes, normalized with respect to the 1‐chloro‐2‐iodoferrocene (**1**), revealed that the obtained trends for the series **1**–**5** are very similar in both cases.

## 2 PLANAR CHIRAL FERROCENES

### 2.1 Recent applications of planar chiral ferrocenes

Among various applications of ferrocenes [62, 88, 89], asymmetric catalysis is by far the domain where planar chirality of substituted ferrocenes was mostly employed. Starting from the 1990s, a huge number of planar chiral ferrocenes were developed as ligands for asymmetric catalysis. Among them, well‐known P,N‐ and P,P‐ligands such as Fc‐Phox (**6**) [90], Josiphos (**7**) [91], Taniaphos (**8**) [92], Walphos (**9**) [93] (Figure S2) have been applied with remarkable asymmetric efficiency, even at industrial level for the production of enantiopure chemicals [94, 95]. Multiple substitution patterns have been used in the preparation of planar chiral ferrocene catalysts, including 1,2‐disubstituted, 1,1′,2‐trisubstituted, 1,1′,2,2′‐tetrasubstituted, polysubstituted and heterocyclic ferrocenes as well as bisferrocenes [21]. As several excellent reviews have been published in this field over time [21, 22, 54, 96], we will report herein only a selection of very recent planar chiral ferrocenes used as organocatalysts or ligands in metal‐catalysed reactions, highlighting the motifs which have been attracting attention of chemists in the last few years in order to expand the scope of ferrocenyl ligands and catalysts [22].

Ferrocene has been considered a privileged framework for the construction of chiral organocatalysts [97]. Very recently, planar chiral catalysts **10**–**12** (Figure S3), featuring ferrocene‐fused nitrogen heterocycles, were developed as systems where the ferrocene backbone provides planar chirality, whereas the heterocyclic moiety maintains its nucleophilic and basic character [98, 99, 100, 101, 102, 103, 104, 105]. Although chiral ferrocenyl catalysts were developed for a long time in asymmetric noncovalent organocatalysis [97], it is not until the last 2 years that our groups reported the first examples of halogen bond (XB)‐based chiral ferrocenyl catalysts [87, 106]. However, only 6% *ee* was obtained with the planar chiral bis‐triazolium catalyst **13** in an aza‐Diels–Alder reaction (Figure S4) [106].

Several examples of planar chiral ferrocenyl phosphines such as monophosphines **14**–**16** were used as ligands in metal‐catalysed reactions, which were recently reported (Figure S5) [107, 108, 109]. Interestingly, Echavarren et al. also reported a series of unusual 1,3‐disubstituted ferrocenyl monophosphines **16** as ligands in gold(I)‐catalysed reactions [109]. Indeed, 1,2‐disubstitution in planar chiral ferrocenes remains a privileged motif, which is prone to function for metal coordination, also through chelating effect. Moreover, most ferrocene ligands and catalysts reported so far combine planar and central chirality, this type of systems affording very positive results in asymmetric catalysis. This trend has also continued in recent years as shown with most recent ferrocenyl monophosphines [110, 111], phosphine phosphoramidite ligands [112], diphosphines [113] and tridentate PNP ligands [114] developed for metal‐catalysis.

**FIGURE 5:**
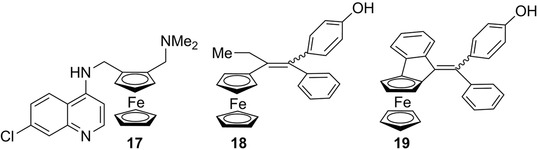
Structures of ferroquine (**17**), ferrocifen (**18**) and planar chiral derivative **19**

Although the impact of planar chirality is not obvious in medicinal chemistry and material science, planar chiral ferrocenes have found important interest also in these fields. Drugs based on the ferrocenyl moiety are attractive because of their good stability, nontoxicity and the ability of ferrocene to generate reactive oxygen species that can induce apoptosis [115]. One of the first described planar chiral ferrocenes with important biological activity is ferroquine **17** (Figure 5), an analogue of the well‐known antimalarial chloroquine [116, 117]. The antimalarial and cell antiproliferative activities of both enantiomers were compared, and no significant differences were found [118]. Afterwards, many other ferroquine analogues were studied for their antimalarial and antiplasmodial activities [119, 120]. Achiral ferrocifen **18** (Figure 5) represents another important class of biologically active compounds initially developed for their structural analogy to tamoxifen, an anti‐tumour drug used in breast cancer therapy [121]. A large number of ferrocifen derivatives with remarkable structural and mechanistic diversity are currently available [61], but only a few being planar chiral [122, 123]. More recently, Guiry et al. described the synthesis of enantiopure analogue **19** of ferrocifen (Figure 5). The biological evaluation of both enantiomers of **19** revealed moderate anticancer activities on breast cancer cells with some difference between the enantiomers [124].

A number of other polycyclic and heterocyclic structures based on planar chiral ferrocenes with interesting biological activities such as compounds **20**–**26** were also reported these last years, which are depicted in Figure S6 [125, 126, 127, 128, 129, 130, 131, 132].

**FIGURE 6:**
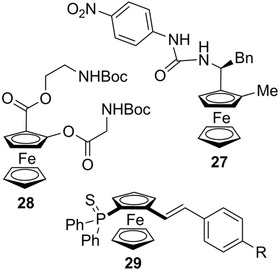
Planar chiral ferrocenes studied in the field of electrochemical sensing

Electrochemical and photophysical properties of ferrocenes combined with planar chirality have resulted in many applications in material sciences. In this field, 1,3‐disubstituted planar chiral ferrocenes were used for the elaboration of optically active liquid‐crystalline polymers [133, 134]. A series of chiral ferrocenic chromophores were also studied for their ability to give non‐linear optic responses in solution and in the solid state [135, 136, 137, 138]. Interestingly, the two planar chiral ferrocenic compounds **27** and **28** were reported to act as electrochemical sensors of chiral dicarboxylates (Figure 6). Although with **27** the combination of central and planar chirality was necessary to obtain good sensing properties [35], interesting results were obtained with the polytopic ligand **28** carrying only planar chirality [139]. Conversely, in a more recent work, chiral ferrocenes of the general structure **29** (Figure 6) were efficiently enantiodifferentiated on chiral polythiophene inherently chiral electrodes [34].

**FIGURE 7:**
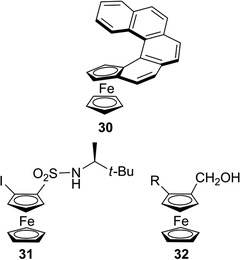
Planar chiral ferrocenes studied as test probes for their chiroptical properties

In the frame of studies on helical chiral molecules that show strong chiroptical properties, the (*R*_p_)‐enantiomer of ferrocene **30** (Figure 7) showed very interesting chiroptical properties, highlighted by exceptional optical rotation value ([*α*]_D_^20^ = +3240) and strong circular dichroism signals [31]. In particular, this planar chiral ferrocene showed additional helical chirality, as a result of a high distortion of the planarity, generating a significant transfer of chirality from the planar chiral ferrocene moiety to the helical *ortho*‐condensed aromatic system [31]. Very recently, experimental ECD and VCD spectra of compounds **31** and **32**, used as test probes, were recorded and studied with the assistance of quantum chemical computations, which allowed for an unambiguous analysis of the different structural factors that may impact the overall spectroscopic features [32, 33] (Figure 7).

### 2.2 Preparation of planar chiral ferrocenes: asymmetric synthesis versus enantioseparation

**FIGURE 8:**
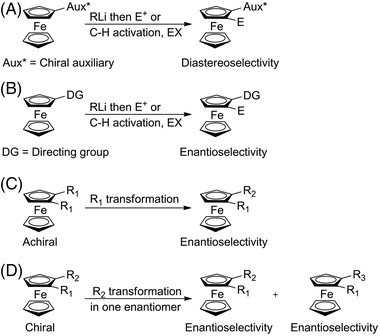
Main approaches for the stereoselective synthesis of planar chiral ferrocenes: diastereoselective methods (A); enantioselective methods (B); desymmetrization of achiral ferrocenes (C); kinetic resolution of chiral ferrocenes (D)

Since the seminal work of Ugi and the revival observed in the early 1990s, three main strategies for the asymmetric synthesis of planar chiral ferrocenes were developed in the literature based (a) on kinetic resolution [140] and desymmetrization methods, (b) on diastereoselective and (c) on enantioselective functionalizations relying on the use of lithiation/electrophilic quenching sequence [25] or metal‐catalysed C–H activation [141]. Diastereoselective methods (Figure 8A) are by far the most employed ones, but the use of chiral auxiliaries is a limiting factor. Indeed, this method involves additional steps to introduce and remove the required substituent. This is the reason why enantioselective methods (Figure 8B) are nowadays more and more targeted. However, the enantiomeric purity of the final products is not as high as the ones obtained through diastereoselective methods. Other very attractive but less explored methods are those based on desymmetrization of achiral ferrocenes (Figure 8C) and on kinetic resolution of chiral ferrocenes (Figure 8D).

Consequently, the number of syntheses of planar chiral ferrocenes is extremely large. However, the search for methods to efficiently obtain pure enantiomers cannot be limited to asymmetric syntheses for the different reasons listed as follows:

- Except for some well‐established diastereoselective methods, it is very rare that the planar chiral ferrocenes are obtained as single enantiomers. Even when very efficient methods are employed, enantiomeric purity over 95% is achieved only in specific cases but most often, depending of the nature of the substituents, it is not sufficiently high.
- Most of the reported asymmetric syntheses give access to only one of both enantiomers.
- There are still some planar chiral ferrocene structures lacking efficient asymmetric synthesis.

**FIGURE 9:**
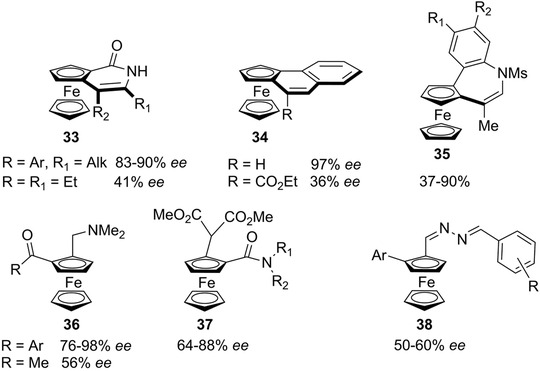
Planar chiral ferrocenes obtained with low to moderate *ees*

**FIGURE 10:**
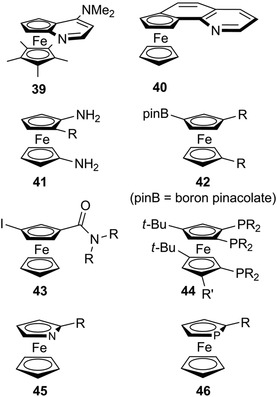
Planar chiral ferrocenes and derivatives with limited availability in their enantiopure forms

These reasons thus justify that more attention should be given to the development of efficient enantioseparation methods. In the following, we will give selected examples illustrating some limitations of asymmetric synthesis for accessing chiral ferrocenes with high enantiomeric purity (Figures 9 and 10):

1. An enantioselective C–H activation/annulation strategy for the synthesis of ferrocene‐fused heterocycles **33** was recently reported [142]. The Rh‐catalysed reaction between ferrocenecarboxamides and internal alkynes afforded the desired compounds, providing high enantiomeric excesses (*ee*s) only when alkyl/aryl nonsymmetric alkynes were used. In contrast, by using the symmetric dialkyl alkyne hex‐3‐yne a as reagent, a low *ee* of 41% was obtained.
2. A Pt‐catalysed reaction was used for the synthesis of planar‐chiral naphthalene‐fused ferrocenes **34** [143]. Although the non‐substituted derivative (R = H) was obtained with 97% *ee*, only a low *ee* of 36% was achieved for the ester‐substituted compound (R = CO_2_Et). A similar strategy was also used for the synthesis of azepine‐fused ferrocene derivatives **35** but the *ee*s were generally moderate [144].
3. The direct Pd‐catalysed asymmetric C–H acylation of 1‐dimethylaminomethylferrocene generated compounds **36** with high *ee*s for insertion of arylketones (R = Ar), but a lower *ee* was obtained in the case of methylketone (R = Me) [40].
4. Several publications reported efficient C–H functionalization methods for the synthesis of planar chiral ferrocenes that generally resulted in moderate *ee*s [145]. The enantioselective alkylation of amidoferrocene with diethylmalonate generated compounds **37** with *ee*s between 64% and 88% [146].
5. A Pd/chiral ligand system was used recently for the double C–H activation of an azine‐based ferrocene to provide heteroaryl functionalized ferrocenes **38** with *ee* in the range 50%–60% [147].
6. As mentioned earlier (see Figure S3), the planar chiral aminopyridine derivatives based on the ferrocene ligands developed by Fu have shown excellent enantioselectivity in a wide range of asymmetric reactions [100, 148]. Interestingly, both enantiomers of **39** could be enantioseparated by HPLC [28]. In two more recent publications by the group of Ogasawara and Yoshida, the asymmetric synthesis of **39** and analogues was realized but only the (*S*_Fc_) enantiomers could be prepared [43, 99]. More extended benzoquinoline derivative **40** was also prepared for catalysis purposes and, again, only the (*S*_Fc_) enantiomer was prepared by asymmetric synthesis [149].
7. The asymmetric synthesis of some relevant planar chiral ferrocene derivatives, such as those functionalized in 1,3‐positions and in both Cp rings, are currently seldom explored in the literature. Planar‐chiral 1,1′‐diaminoferrocenes **41** were recently prepared as racemates, which are key substrates for the synthesis of chiral *N*‐heterocyclic carbenes and tetrylene analogues [150]. On the other hand, the synthesis of enantioenriched or enantiopure 1,3‐disubstituted ferrocenes is much more difficult compared to the 1,2‐disubstituted one, because the strategies relying on directed functionalization cannot be applied. For instance, enantioenriched and enantiopure borylated planar‐chiral ferrocenes, which represent important building blocks for the synthesis of more functionalized chiral compounds, were described for accessing 1,2‐disubstituted derivatives [42, 151], whereas no asymmetric method is currently available for 1,3‐disubstituted ferrocenes **42** [152]. Analogously, several asymmetric methods are available for the synthesis of 1‐iodo‐2‐amidoferrocenes, but no direct methods were described for the synthesis of enantioenriched or enantiopure 1,3‐disubstituted derivatives **43**, for which an efficient racemic synthesis was described [153], exclusively.
8. In the last decade, the Hierso group reported several planar chiral polyphosphine derivatives **44** as efficient ligands in coordination chemistry [154, 155, 156], but because of their structural complexity, no asymmetric synthesis was explored.
9. Two heteroatom‐containing ferrocenes, namely azaferrocene **45** and phosphaferrocene **46**, represent important analogues of ferrocene with reported applications in asymmetric catalysis [157]. These derivatives are planar chiral when only one position of the heterocyclic ring is functionalized and several derivatives could be enantioseparated by HPLC [148, 158]. However, their preparation in enantiopure forms through asymmetric synthesis remained scarce. The asymmetric synthesis of planar chiral azaferrocenes relied either on the *n*‐BuLi/sparteine strategy to deprotometallate enantioselectively one α‐position [159, 160], or on the Kagan's chiral sulfoxide strategy [161, 162]. Enantiopure phosphaferrocenes were generally obtained by a prior synthesis of chiral phospholides [163, 164], and their direct enantioselective formation was reported only in one instance [165].

The examples mentioned before contributed to profile a scenario where liquid‐phase enantioseparation still may play a pivotal role for accessing planar chiral ferrocene in enantiopure or enriched form. Despite that, so far limited attention has been devoted to this issue by scientists operating in the field of enantioseparation science.

## 3 ENANTIOSEPARATION OF PLANAR CHIRAL FERROCENES

**FIGURE 11:**
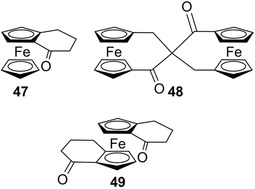
Structures of ferrocene derivatives **47**–**49**

In the late 1950s, most methods for obtaining enantiopure chiral ferrocenes relied on chemical resolution through diastereoselective crystallization. In 1959, Thomson reported the resolution of the first chiral ferrocene derivative, namely, ferrocenocyclohexenone **47** (Figure 11), by diastereomeric crystallization [166].

**FIGURE 12:**
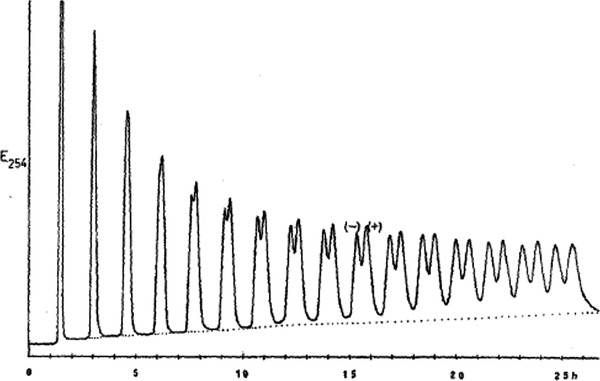
Chromatographic resolution of ferrocene **49** in 16 cycles under medium‐pressure conditions. *Source*: Reprinted from Ref. [168] with permission

Later, the first chromatographic resolution of a ferrocene derivative was reported by Schlögl, who enantioseparated compound **47** on acetylated cellulose [15]. The partial enantioseparations of ferrocenes **48** [167] and **49** [168] (Figure 11) were also obtained by the Schlögl's group on partially acetylated cellulose, with benzene as mobile phase, and on microcrystalline triacetylcellulose with ethanol, respectively. In particular, at that time, the enantioseparation of **49** provided poor results after only one run under medium‐pressure chromatographic conditions. Thus, the partial enantioseparation required 16 cycles by using a recycling technique, although, after about 15 runs on a 30‐cm column, the separation window got saturated due to the peak broadening, so that no further increase of enantiomeric purity could be achieved (Figure 12).

In 1985, Armstrong et al. reported the first successful enantioseparations of chiral ferrocenes by enantioselective HPLC [49]. In this study, 11 ferrocenes containing a chiral centre, as stereogenic unit, were enantioseparated on a β‐cyclodextrin (CD)‐bonded CSP by using methanol (MeOH)/water mixtures as mobile phases, obtaining *α* values ranging from 1.06 to 1.39. It is worth mentioning that in the early 1980s, several nonchromatographic studies by Breslow [169, 170, 171] and Takahashi [172] also reported that chiral recognition was possible between CDs and various enantiomeric ferrocene compounds. Later, other CD‐based CSPs were used for the enantioseparation of ferrocenes featured by central chirality exclusively [51, 173–175]. Recently, sulfobutylether‐β‐CD was used as a chiral selector for the CE enantioseparation of chiral ferrocenes exclusively containing a chiral centre, as a chiral element [176].

**FIGURE 13:**
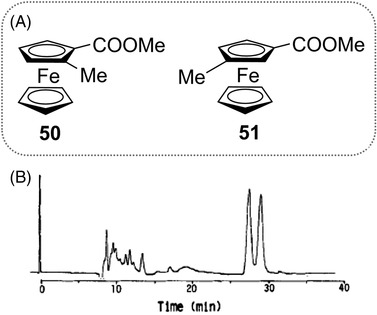
Structures of ferrocenes **50** and **51** (A), and enantioseparation of ferrocene **50** (B). Column, Cyclobond I; mobile phase, 47% MeOH/water; flow rate, 0.4 ml/min. *Source*: Adapted from Ref. [177] with permission

In 1989, the first HPLC enantioseparations of planar chiral ferrocenes **50** and **51** (Figure 13A) were reported by Yamazaki et al. by using a β‐CD‐bonded column and a 47% MeOH/water mixture as mobile phase [177]. The enantioseparation of the ferrocene derivative **50** is reported in Figure 13B. Under the same elution conditions, the enantiomers of ferrocene **51** were eluted with retention times of 32.5 and 33.9 min, respectively. In 1991, the same group reported the first HPLC enantioseparations of planar chiral ferrocenes (compounds **50**–**55**) on a polysaccharide‐based chiral column, the Chiralcel OD containing cellulose *tris*(3,5‐dimethylphenylcarbamate) as chiral selector coated in silica gel, by using a *n*‐hexane/2‐propanol (2‐PrOH) mixture as a mobile phase [178] (Table 1). Under these conditions, ferrocenes **54** and **55** were not eluted.

**TABLE 1:** First enantioseparations of planar chiral ferrocenes **50**–**55** on Chiralcel OD (*n*‐hexane/2‐PrOH mixture) [178]

Fc	*k*_1_ ^a^	*k*_2_ ^a^	*α* ^b^	*R*_s_ ^c^	EEO^d^
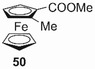	0.63	0.88	1.39	2.94	*R*_p_–*S*_p_
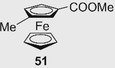	0.99	1.35	1.35	3.41	*R*_p_–*S*_p_
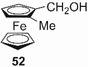	2.27	2.58	1.14	1.81	*S*_p_–*R*_p_
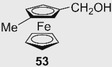	1.77	1.99	1.12	1.46	*R*_p_–*S*_p_
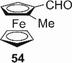	Not eluted				
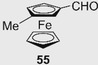	Not eluted				

^a^ Retention factor.

^b^ Selectivity factor.

^c^ Resolution.

^d^ Enantiomer elution order.

Given the versatility and the remarkable load capacity of the polysaccharide‐based CSPs, in the second half of the 1990s, the first semipreparative HPLC enantioseparations of planar chiral ferrocenes were successfully performed by the Fu's group by using, in most cases, Chiralcel OD as a chiral column (Figure S7). In 1996, the enantioseparation of the planar chiral azaferrocene **56** with a Chiralcel OD column and *n*‐hexane/2‐PrOH 90:10 v/v as mobile phase [148] was performed, recovering each enantiomer with 98% *ee*. A year later, the same group accessed pure enantiomers of azaferrocene **57** [179], and planar chiral 4‐(dimethylamino)pyridine analogues **39** and **58** [28] again on Chiralcel OD, by using *n*‐hexane/2‐PrOH 90:10 v/v, *n*‐hexane/2‐PrOH/diethylamine (DEA) 50:50:0.2 v/v/v and *n*‐hexane/2‐PrOH/DEA 75:25:0.4 v/v/v, respectively, as mobile phases. In 1998, pure enantiomers of the *C*_2_‐symmetric bisazaferrocene **59** were also recovered on Chiralcel OD (*n*‐hexane/2‐PrOH/DEA 95:5:0.1 v/v/v) with >99% *ee* [158]. Then, the semipreparative enantioseparation of phosphaferrocene **60** was also performed on the Chiralcel OD (*n*‐hexane/2‐PrOH 90:10 v/v) [180]. It is worth mentioning that, later, the Fu's group also reported about the semipreparative enantioseparation of a *C*_2_‐symmetric planar chiral bipyridine ligand with the brush‐type column Whelk‐O1 [181]. In the same period, Bolm et al. reported the first utilization of an amylose‐based column, the Chiralpak AD, for the semipreparative enantioseparation of a planar chiral ferrocene, obtaining the pure enantiomers of the ferrocene‐based hydroxyloxazoline **61** with >99.8% *ee* under normal phase conditions [26].

**FIGURE 14:**
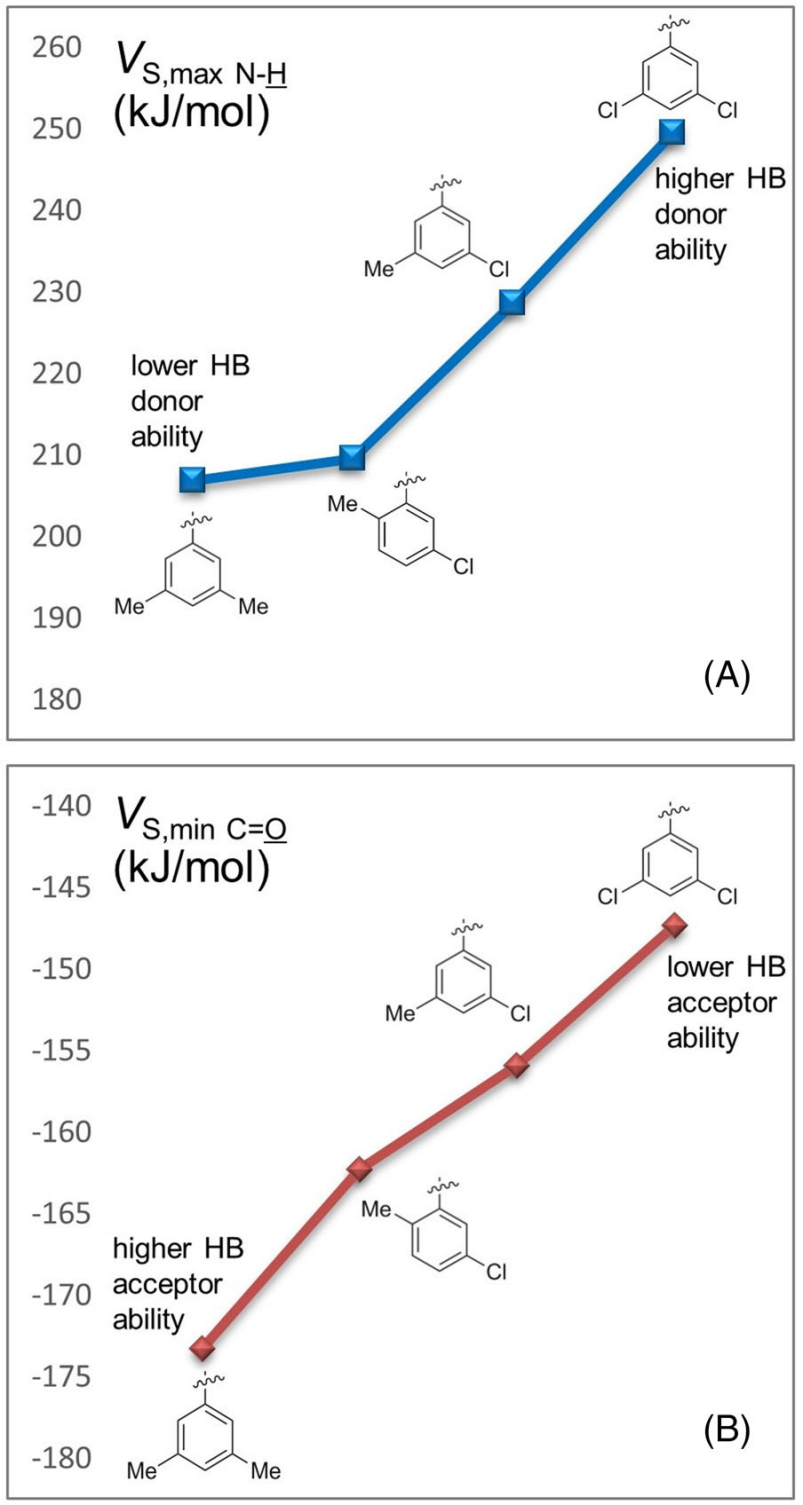
Comparison of *V*_S,max_ (A) and *V*_S,min_ (B) calculated for the amidic hydrogens and the carbonyl oxygens, respectively, on isodensity surfaces, for the 3,5‐dimethyl‐, 5‐chloro‐2‐methyl‐, 3‐chloro‐5‐methyl‐ and 3,5‐dichlorophenylcarbamates as pendant groups

Despite the fact that the first enantioseparations of planar chiral ferrocenes were performed on a CD‐based CSP [177], already at the dawn of planar chiral ferrocene enantioseparations polysaccharide‐based CSPs emerged as privileged platforms to enantioseparate this class of organometallic compounds. As a result, in the last decades the enantioseparations of planar chiral ferrocenes have been performed by using polysaccharide‐based CSPs almost exclusively, and just few enantioseparations with other types of chiral selectors were reported. In this regard, it is worth mentioning that the key structural elements of these polymeric selectors are the backbone (amylose or cellulose), and the pendant groups (phenylcarbamates or esters) which synergistically determine the distinctive resolving ability of the corresponding chiral columns (Table S2). The main interactions involving phenylcarbamate and ester (benzoate or cinnamate) pendant groups of these selectors are HBs, dipole–dipole and π–π interactions, along with XBs and chalcogen bonds [182]. However, these latter two types of interactions only participate in binding and recognition of analytes with specific structural requirements [183]. In particular, the methyl or/and chlorine substituents on the phenyl rings of the pendant groups modulate the electron charge density on the O=C–N–H moieties of the polymers, determining the strength of the carbonyl oxygens, as HB/XB acceptors, and of the amidic hydrogens, as HB donors. In Figure 14, for some representative polysaccharide‐based selectors featuring chiral columns used in the enantioseparation of planar chiral ferrocenes over time (Table S2), the HB capability of the corresponding O=C–N–H moieties was compared by changing the distinctive substitution on the phenyl rings of the pendant groups. This comparison was performed in terms of the associated *V*_S,max_ and *V* minima (*V*_S,min_) calculated respectively for the amidic hydrogens and the carbonyl oxygens on isodensity surfaces [183].

In the next sections, enantioseparations of planar chiral ferrocenes performed on polysaccharide‐based CSPs in the last decades will be described. For clarity, names and features of the chiral columns mentioned in the following sections are summarized in Table S2. The reported procedures will be categorized on the basis of the structural features of the analytes, determined in turn by the distinctive substituents bound to the ferrocene moiety: (a) polar groups such as OH, C=O, COOH and COOR and others containing O and N atoms (Section 3.1), (b) substituents containing extended π‐electronic clouds (Section 3.2) and (c) halogen atoms (Section 3.3). This choice is based on the fact that properties, functions and mechanisms of chiral chromatographic systems are based on concepts at the interface of analytical, physical and organic chemistry. Indeed, on the one hand, the enantioseparation process concerns the adsorption phenomenon underlying retention mechanism of analyte molecules which compete with solvent molecules onto the CSP surface [182]. On the other hand, chiral analytes, selectors and most mobile phase components are organic compounds. As a consequence, the structural properties (shape, geometry and electronic distribution) of the three pivotal components of the chromatographic system play a key role in the enantiodistinction processes. Considering these features may help analytical scientists to design enantioseparation procedures by evaluating steric and electronic properties of the molecular structures involved in the chromatographic system, going beyond trial‐and‐error approaches for methods development.

### 3.1 Planar chiral ferrocenes containing polar groups

**FIGURE 15:**
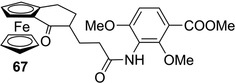
Structures of ferrocenocyclohexenone **67**

**FIGURE 16:**
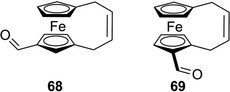
Structure of the bridge‐substituted [3]ferrocenophanes **68** and **69**

Given that planar chiral ferrocenes with coordinative properties have attracted great interest for applications in several fields, most HPLC enantioseparations of planar chiral ferrocenes performed over time concern ferrocenes containing polar groups such as NH_2_, NHR, NRR_1_, C=O, COOH, COOR and OH. Following the pioneering studies of Schlögl [168] and Yamazaki [177], in 2002 the first analytical study on enantioseparation of ferrocene containing the carbonyl moiety as distinctive group was reported by Buchmeiser et al. [44]. In this study, the enantioseparation of planar chiral ferroceno[2,3‐a]inden‐1‐ones and derivatives was explored by using five homemade β‐CD‐based silica supports under polar organic conditions (ACN/MeOH/acetic acid/triethylamine [TEA] 99:1:0.1:0.1 v/v/v/v, *T* = 0°C). The carbonyl moiety proved to be essential for achieving successful enantioseparation. Indeed, whereas ferroceno[2,3‐a]inden‐1‐one (**62**) and derivatives could be enantioseparated with 1.62 ≤ *α* ≤ 2.15, ferroceno[2,3‐a]indene (**63**) and alkoxyferroceno[2,3‐a]indene (**64**) were not resolved. These first observations highlighted that the enantioseparation of nonpolar chiral planar ferrocenes may be rather challenging, this fact representing a still open issue. Again in 2002, in a paper reporting the synthesis of ferroquine enantiomers, Delhaes et al. mentioned the HPLC analytical enantioseparation of the 2‐(*N*,*N*‐dimethylaminomethyl)ferrocenecarboxaldehyde (**65**) and of the 2‐(*N*,*N*‐dimethylaminomethyl)ferrocenylmethyl acetate (**66**) by using the Chiralcel OD [118]. However, no other information was provided by the authors about these analyses. In 2011, Metzler‐Nolte et al. reported the separation of the enantiomers (*R*,*R*_p_) and (*S*,*S*_p_) of the ferrocenocyclohexenone **67** (Figure 15) on Chiralpak IA under normal phase conditions (*n*‐hexane/2‐PrOH 60:40 v/v) [59]. Ferrocene **67**, featuring central and planar chirality, was prepared as intermediate for the synthesis of chiral ferrocene‐containing platensimycin derivatives with potential antimicrobic activities. Recently, Buchowicz et al. reported the analytical enantioseparation of the bridge‐substituted [3]ferrocenophanes **68** and **69** (Figure 16) used as synthetic intermediates for the synthesis of potential anticancer metallocenes [128]. For the purpose, Chiralcel OD‐H and Chiralpak AD‐H were used as chiral columns for the enantioseparation of **68** and **69**, respectively, the 1,3‐disubstituted derivative being enantioseparated on the cellulose‐based column (*n*‐hexane/2‐PrOH 95:5) better compared to the 1,2‐disubstituted analogue on the Chiralpak AD‐H (*n*‐hexane/EtOH 98:2). This couple of enantioseparations highlighted the fact that 1,2‐disubstituted ferrocenes are often enantioseparated with lower *α* compared to the 1,3‐disubstituted systems, likely due to the steric hindrance occurring between the two adjacent functional groups which may hinder selector–selectand interactions.

In several asymmetric synthesis studies, the determination of the enantiomeric purity of large series of structurally related planar chiral ferrocenes was performed. On the one hand, the evaluation of these chromatographic outcomes may allow for gaining information about structure–chromatographic behaviour relationships, accounting for the impact of analyte and selector structure, and of mobile phase polarity. Indeed, the chromatographic parameters, as well as the enantiomer elution order (EEO), are the macroscopic expressions of the enantioseparation process occurring at molecular level. In other words, they are signs of the noncovalent interaction between selector and selectand. On the other hand, in practice, only attempts to derive tentative guidelines for the enantioseparation of further planar chiral ferrocenes can be made given that a great variability of chromatographic conditions often occurs within these series of enantioseparations, and the systematic evaluation of the impact of a single factor may not be easy to derive.

In 2014, Cui et al. reported the first catalytic and enantioselective C–H direct acylation of ferrocene derivatives affording 17 2‐acyl‐1‐dimethylaminomethylferrocenes (**36a**–**q**) with 56% ≤ *ee* ≤ 98% [40]. Determination of the enantiomeric purity was performed through enantioselective HPLC by using amylose‐based Chiralcel IE‐3 and cellulose‐based Chiralcel IC‐3 (Table S3), as chiral columns, both containing the 3,5‐dichlorophenylcarbamate as a pendant group. By evaluating the chromatographic outcomes of ferrocenes **36a**–**q**, some remarks can be made:

1. Given the structure of analytes and selectors, it is likely that binding and recognition mechanisms are governed by HB occurring between the carbonyl oxygen of the analyte and the N–H of the selector pendant group, which is equal in both columns and with the highest HB donor ability (Figure 14).
2. The HB acceptor ability of the analytes is affected by the R substituent of the carbonyl moiety which modulates the electron charge density on the carbonyl oxygen and the steric hindrance of the neighbouring space. For instance, for the enantioseparations on the Chiralpak IC‐3, both electronic and steric effects appeared to impact the enantioseparation. Indeed, under the same conditions, the enantioseparation of **36n** (R = Et) was lower than that of **36** **m** (R = Me), likely due to a negative steric impact of the alkyl chain. Then, the presence of the chlorine, as an electron‐withdrawing substituent, decreases the enantioseparation extent for **36o** and **36p** compared to the methylated compound **36q** (R = 3,4‐dimethylphenyl). A similar effect also occurred on the Chiralpak IE‐3. Indeed, considering compound **36c** (R = Ph) as reference for comparison, both ferrocenes **36a** and **36b**, containing strong electron‐withdrawing groups such as F and CF_3_, showed lower enantioseparation, the latter providing the lowest enantioseparation within the series **36**.
3. The EEO was always *R*_p_–*S*_p_ on the amylose‐based column, whereas a backbone‐dependent EEO reversal (*S*_p_–*R*_p_) occurred on the Chiralpak IC‐3. A single exception to this trend could be observed for the enantiomers of compound **36i** which were eluted on the Chiralpak IE‐3 with the order *S*_p_–*R*_p_. In this regard, it could be noted that, among all compounds enantioseparated on the Chiralpak IE‐3, only **36i** contains a furanyl group, as a distinctive substituent at the carbonyl moiety. This unit is more polar compared to the hydrophobic phenyl ring featuring all other derivatives and likely induces a mechanism change as revealed by the observed EEO reversal.

Later, You et al. reported the asymmetric synthesis of 22 planar chiral pyridine ferrocenopentadienone derivatives (**70a**–**v**) via Pd‐catalysed intramolecular C−H arylation with high enantioselectivity (96%–99% *ee*), determining the enantiomeric purity of the prepared compounds by HPLC on polysaccharide‐based Chiralcel OD‐H, Chiralcel OJ‐H, Chiralpak AD‐H and Chiralpak AS‐H, as chiral columns, with *n*‐hexane/2‐PrOH mixtures as mobile phases (Table S4) [29]. For these enantioseparations, some observations can be made as follows:

1. Twelve compounds (**70a**–**l**) were enantioseparated on Chiralcel OD‐H with *n*‐hexane/2‐PrOH 90:10 v/v as mobile phase. Given compound **70a** as reference for comparison, it can be noted that structural variations of the groups R on the pyridine ring (**70i** and **70j**), or of R_1_ on the other Cp ring (not containing the carbonyl group) (**70c**–**g**), or of both (**70k** and **70l**) do not produce EEO reversal, and in all these cases the enantiomers eluted as *S*_p_–*R*_p_. Otherwise, for compounds **70b** and **70h** showing structural variations at the two ‘external’ positions of the pyridine ring, an EEO reversal (*R*_p_–*S*_p_) occurred. Interestingly, despite the structural differences of the corresponding selectors, the same trend could be observed on the Chiralpak AD‐H and on the Chiralpak AS‐H for compounds **70o** and **70v**, respectively, showing *R*_p_–*S*_p_ as EEO associated with structural variations of the external part of the pyridine rings. Otherwise, compounds which did not present this feature such as **70n**, **70p**, **70q** and **70s**–**u** provided *S*_p_–*R*_p_ as EEO.
2. On the Chiralcel OD‐H, the presence of fluorine as a substituent on the pyridine ring (**70l**: R = F, *t*_R_ = 7.7 min and 11.0 min) had a detrimental effect on retention and on the enantioseparation extent compared to the analogue containing an electron‐donating group at the same position (**70k**: R = NMe_2_, *t*_R_ = 10.9 and 21.2 min). On this column, the steric hindrance of the substituent R_1_ impacted retention and selectivity. Thus, in compound **70f**, the presence of the bulky CHMe_2_OH (*t*_R_ = 12.0 and 14.0 min) induced a decrease of retention compared to the analogue **70e** (*t*_R_ = 24.3 and 28.1 min). Otherwise, on Chiralpak AD‐H, changing the methyl groups at the pentasubstituted Cp ring (**70p**: R_1_ = Me, *t*_R_ = 5.5 and 5.8 min) to the ethyl groups (**70q**: R_1_ = Et, *t*_R_ = 5.3 min and 6.4 min) decreased and increased the retention of the first and the second eluted enantiomers, respectively, and as a result, the enantioseparation extent increased.

In 2020, Guiry et al. reported the enantioseparation of the ferroceno[2,3‐a]inden‐1‐one (**62**) under supercritical fluid chromatography (SFC) conditions (Chiralpak IB, scCO_2_/2‐PrOH 99:1 to 60:40 v/v over 7 min, EEO = *S*_p_–*R*_p_) for determining the enantiomeric purity (99% *ee*) of the compound used as intermediate for the synthesis of planar chiral ferrocifens [124].

**FIGURE 17:**
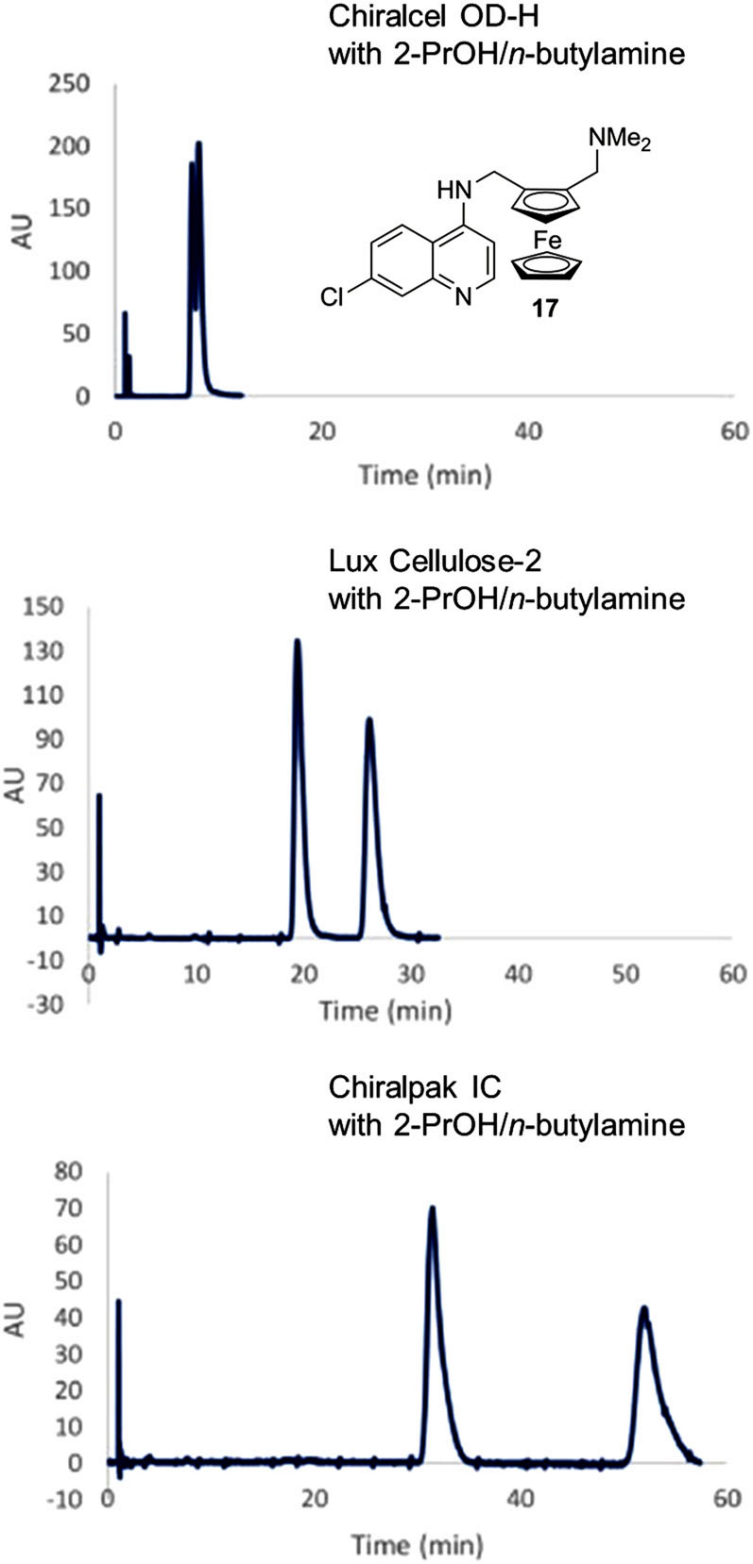
Chromatograms obtained for the enantiomers of compound **17** on Chiralcel OD‐H, Lux Cellulose‐2 and Chiralpak IC with 30% 2‐PrOH and 1% of *n*‐butylmine in carbon dioxide, at a flow‐rate of 3 ml/min, column temperature of 40°C, 150‐bar outlet pressure, *λ* = 260 nm. *Source*: Adapted from Ref. [48] with permission

Very recently, a first analytical study on the enantioseparation of planar chiral ferrocenes in SFC was reported by Lipka et al. [48]. In this study, the authors analysed several chiral metallocenes (M = Fe, Ru), among them five 1,2‐ and 1,3‐disubstituted planar chiral ferrocenes, by using eleven polysaccharide‐based CSPs (Lux Cellulose‐2, Amylose‐2 and Amylose‐3, Chiralcel OD‐H and OJ‐H, Chiralpak IB, IC, AD‐H, AS‐H, IA and IG) with carbon dioxide containing 30% of MeOH or 2‐PrOH as a co‐solvent in the mobile phase. Among the planar chiral ferrocenes, one analyte contained two carbonyl groups, two derivatives featured amino moieties, and diphosphine and phosphamino derivatives were also included in the series. The elution of the analytes was optimized by adding 1% basic additive, such as DEA, TEA or *n*‐butylamine to the mobile phase. In most cases, planar chiral ferrocenes involved in this study showed high retention and selectivity factors on the chlorinated cellulose‐based CSPs which exhibited the highest rate of success compared to the non‐chlorinated chiral supports. For instance, a resolution value equal to 14.1 was obtained for the enantioseparation of ferroquine **17** on the Chiralpak IC, based on the cellulose tris(3,5‐dichlorophenylcarbamate) as a chiral selector, by using 30% 2‐PrOH and 1% *n*‐butylamine, whereas poor resolution was observed under the same conditions on the Chiralcel OD‐H containing cellulose *tris*(3,5‐dimethylphenylcarbamate) as a chiral selector (Figure 17). Moreover, the study evidenced interesting performances of the benzoate‐type Chiralcel OJ‐H towards the 1‐[(1*R*)‐1‐(dicyclohexylphosphino)ethyl]‐2‐(diphenylphosphino)ferrocene **71** containing phenyl and cyclohexyl groups, as substituents of the P atom, which are able to exert π‐π and hydrophobic interactions. This compound showed selectivity (*α* = 1.51) and resolution factors (*R*_s_ = 3.58) on this column higher compared to the more polar **17** (*α* = 1.32, *R*_s_ = 1.57). This study paves the way for interesting applications of SFC for the enantioseparation of planar chiral ferrocenes, in particular for preparative purposes.

**FIGURE 18:**
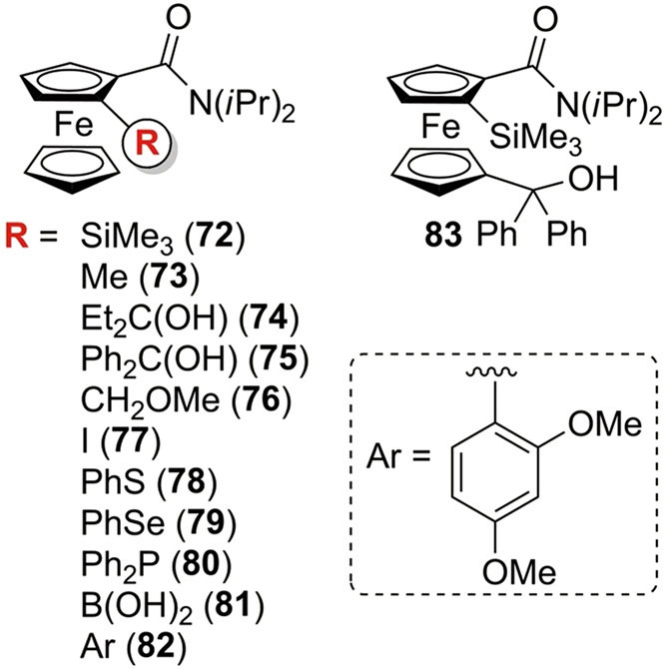
Structures of 1,2‐substituted *N*,*N*‐diisopropyl ferrocenecarboxamides **72**–**82** and of the 1,1′,2‐trisubstituted derivative **83**

Several enantioseparations for analytical purposes were reported involving planar chiral ferrocenes featured by the O=C–N– moiety. In 1996, Snieckus et al. reported the HPLC enantioseparation of 12 substituted *N*,*N*‐diisopropyl ferrocenecarboxamides **72**–**83** (Figure 18) in order to establish the *ee*s of the products prepared by asymmetric synthesis [37]. In this case, Chiralcel OD, OK and OJ were used as chiral columns. Almost all enantioseparations were performed by using *n*‐hexane‐based mixtures as mobile phases, with the exception of ferrocenes **73** and **81** which were enantioseparated by using aqueous–alcoholic mixtures (Table 2). Later, the same group reported the analytical enantioseparation of planar chiral *N*‐cumyl‐*N*‐ethylferrocenecarboxamide **84**, *N*‐ethylferrocenecarboxamides **85a**–**f**, methoxycarbonylferrocenes **86a**–**c**, ferrocenylpiperidinone **87**, ferrocenyldihydroazepinone **88** and ferrocenyltetrahydroazepinone **89** by using the Chiralcel OD, as chiral column, and *n*‐hexane/2‐PrOH mixtures as mobile phases (Table S5) [184]. In 2007, Whiting et al. reported the analytical enantioseparation of the analogue 2‐bromo‐(*N*,*N*‐diisopropyl)ferrocenecarboxamide (**90**) on Chiralcel OD by using *n*‐hexane/ether/DEA 80:20:0.5 v/v/v as mobile phase [151].

**TABLE 2:** Enantioseparation conditions of planar chiral ferrocenes **72**–**83** (Figure 18) on cellulose‐based chiral stationary phases (CSPs) [37]

Fc	Chiral column	Mobile phase	*T* (°C)	Flow rate (ml/min)
**72**	Chiralcel OD	1.5% *t*BuOMe in *n*‐hexane	40	1.5
**73**	Chiralcel OJ	5% Water in ethanol (EtOH)	5	0.2
**74**	Chiralcel OD	10% (Et_2_O/Et_2_NH 0.5%) in *n*‐hexane	rt^a^	1.0
**75**	Chiralcel OD	3% (Et_2_O/Et_2_NH 0.5%) in *n*‐hexane	rt	1.5
**76**	Chiralcel OD	10% (Et_2_O/Et_2_NH 0.5%) in *n*‐hexane	rt	0.9
**77**	Chiralcel OD	15% (Et_2_O/Et_2_NH 0.5%) in *n*‐hexane	rt	1.0
**78**	Chiralcel OD	2% 2‐PrOH in *n*‐hexane	rt	0.5
**79**	Chiralcel OD	2% 2‐PrOH in *n*‐hexane	rt	0.5
**80**	Chiralcel OD	10% (Et_2_O/Et_2_NH 0.5%) in *n*‐hexane	rt	1.5
**81**	Chiralcel OK	5% Water in MeOH	rt	1.0
**82**	Chiralcel OD	2% (Et_2_O/Et_2_NH 0.5%) in *n*‐hexane	rt	1.5
**83**	Chiralcel OD	8% (Et_2_O/Et_2_NH 0.5%) in *n*‐hexane	rt	1.5

^a^ Room temperature.

Very recently, large series of planar chiral alkylated ferrocene carboxamides **37a**–**h** and **91**–**97** (Table S6) [146], borylated ferrocenes **98a**–**y**, **99a**–**f** and **100**–**102**, and ferrocene carboxamide **103** (Table S7) [42], and ferrocene‐fused pyridones **33a**–**u**, **104a**–**k** and **105** (Table S8) [142] were analytically enantioseparated on polysaccharide‐based chiral columns under normal phase conditions. For compounds **98** and **99**, in most cases, Chiralpak IE and IC were used as chiral columns, in general, the latter providing lower retention times compared to the amylose‐based column. Interestingly, compounds of the series **33**, **104** and **105** showed exceptional high optical rotation values up to [*α*]_D_^20^ = +3665.

In the last few years, planar chiral ferrocenes containing the ureido moiety were enantioseparated on polysaccharide‐based CSPs. Tucker et al. determined the enantiomeric purity of the *N*‐(4‐nitrophenyl)‐*N*′‐[2‐methyl‐ferrocenemethyl]‐urea **106** on Chiralpak AD (*n*‐hexane/2‐PrOH 90:10 v/v) [35]. Later, the same group reported the preparation of a series of hydroxyalkyl ferrocene‐nucleosides (**107**) in order to evaluate their potential anticancer activity [131]. The enantiomers of these compounds were characterized by a combination of chiral analytical HPLC and single‐crystal X‐ray diffraction. In this frame, the enantiomeric purity of ferrocenes **107** was determined by using Lux Cellulose‐1 as chiral column with 30% ACN in water as mobile phase. More recently, Erb et al. reported the enantioseparation (Chiralpak IC‐3, *n*‐hexane/2‐PrOH 90:10 v/v) of the 4‐(4‐(3‐(2‐chloroferrocenyl)ureido)‐3‐fluorophenoxy)‐*N*‐methylpicolinamide **108** in the frame of a study on synthesis and biological evaluation of regorafenib analogues and their ferrocenic derivatives [132].

**FIGURE 19:**
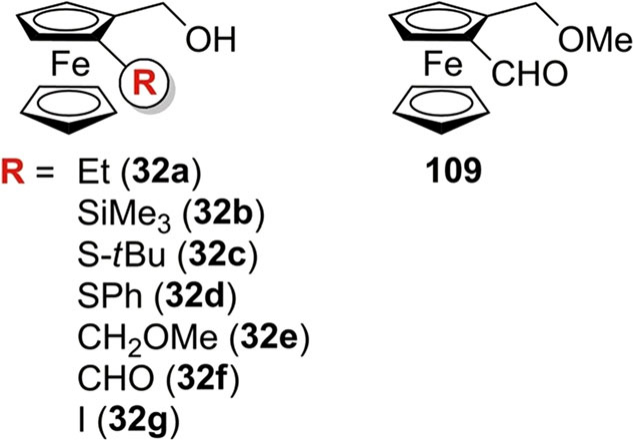
Structures of 1,2‐substituted hydroxymethyl planar chiral ferrocenes **32a**–**g** and the 1‐formyl‐2‐methoxymethylferrocene (**109**)

**FIGURE 20:**
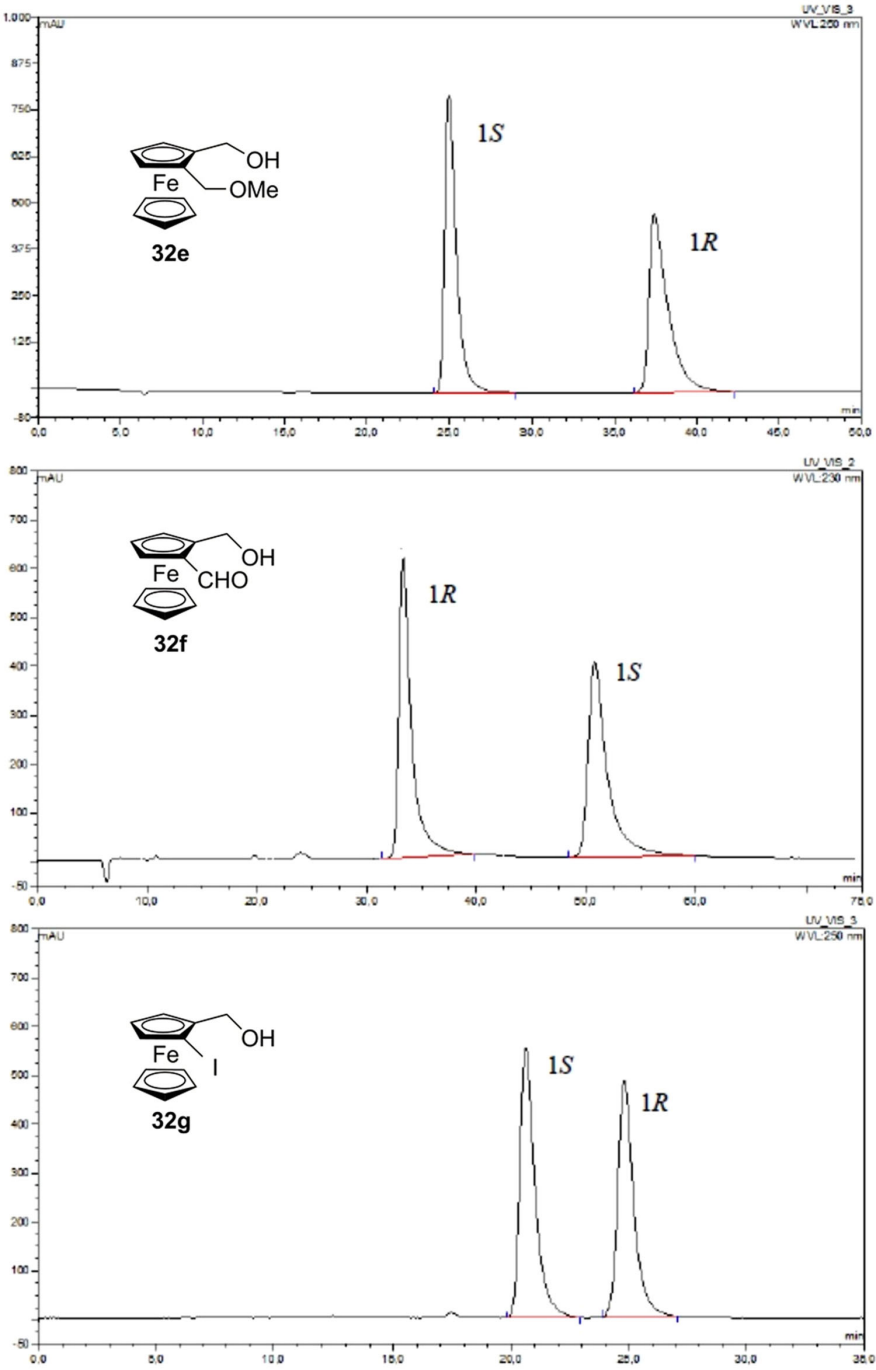
Chromatographic traces of the high‐performance liquid chromatography (HPLC) enantioseparation of compounds **32e**–**g** on Lux Cellulose‐2 (see Table 11 for elution conditions). *Source*: Adapted from Ref. [33] with permission

Over time, enantioseparations of planar chiral ferrocenes featured by hydroxyl groups were also described. Patti et al. reported the HPLC enantioseparation of 1,2‐substituted hydroxymethyl ferrocenes **32a**–**d** [50] and **32e**–**g** [33], and of 1‐formyl‐2‐methoxymethylferrocene (**109**) [33] on cellulose‐based CSPs (Figure 19). The enantioseparations of ferrocenes **32a**–**d** were comparatively studied on Chiralcel OD and Chiralcel OJ as chiral columns under normal phase elution conditions [50]. All the alcohols **32a**–**d** were poorly retained and not sufficiently resolved on the Chiralcel OD, which appeared not suitable as chiral column for this type of analytes. On Chiralcel OJ, both **32a** and **32c** were eluted as single peaks, even with *n*‐hexane/2‐PrOH 99:1 v/v as mobile phase. Otherwise, a sufficient degree of enantioseparation was achieved for **32b** (*n*‐hexane/2‐PrOH 93:7 v/v: *k*_2_ = 1.04, *α* = 1.23, *R*_s_ = 1.62) and **32d** (*n*‐hexane/2‐PrOH 90:10 v/v: *k*_2_ = 13.14, *α* = 2.88, *R*_s_ = 12.70) on Chiralcel OJ, the latter ferrocene being resolved with higher *α* and *R*_s_ values. This result showed the pivotal role of the phenyl ring in the enantiodifferentiation process of this class of planar chiral ferrocenes on Chiralcel OJ. Very recently, in the course of a spectroscopic investigation on the chiroptical properties of ferrocenes **32e**–**g** and **109**, the same group performed the enantioseparation of these analytes by using the chlorinated Lux Cellulose‐2, as chiral column, and *n*‐hexane/2‐PrOH mixtures, as mobile phases (Table 3 and Figure 20) [33]. It is worth noting that changing polar groups such as –CH_2_OMe and –CHO, as substituents of the hydroxymethylferrocene scaffold (compounds **32e** and **32f**, respectively), to the hydrophobic iodine, featuring ferrocene **32g**, had a detrimental effect on the enantioseparation in terms of selectivity (Figure 20). Indeed, as mentioned earlier, the enantioseparation of ferrocenes containing nonpolar substituents exclusively is actually more challenging compared to that of polar ferrocenes.

**TABLE 3:** Enantioseparation of planar chiral ferrocenes **32e–g** and **109** on Lux Cellulose‐2 (mobile phase, *n*‐hexane/2‐PrOH mixtures; flow rate, 0.5 ml/min) [33]

Fc	*n*‐Hexane:2‐PrOH (v/v)	*t*_R1_ (min)^a^	*t*_R2_ (min)^a)^	EEO^b^
**32e**	85:15	25.01	38.53	*S*_p_–*R*_p_
**32f**	75:25	33.92	51.52	*R*_p_–*S*_p_
**32g**	90:10	20.62	24.81	*S*_p_–*R*_p_
**109**	75:25	19.58	23.91	*R*_p_–*S*_p_

^a^ Retention time.

^b^ Enantiomer elution order.

The same group reported the enantioseparation of the planar chiral 1‐acetyl‐2‐methoxymethylferrocene (**110**) on the Chiralcel OD by using *n*‐hexane/2‐PrOH 90:10 v/v as mobile phase [185]. In the same study, two ferrocenes containing both central and planar chirality were also enantioseparated under the same conditions, 1‐acetoxyethyl‐2‐methoxymethylferrocene (**111**) (*t*_R_ = 9.7 min (1*R*_p_,*S*) and 16.3 min (1*S*_p_,*R*)) and the 1‐hydroxyethyl‐2‐methoxymethylferrocene (**112**) (*t*_R_ = 15.5 min (1*R*_p_,*S*) and 24.5 min (1*S*_p_,*R*)). In both cases, the second diastereoisomer could not be resolved under the reported chromatographic conditions.

**FIGURE 21:**
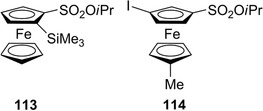
Structures of the isopropyl ferrocenesulfonate **113** and **114**

Very recently, ferrocene sulfonates have attracted interest as synthetic intermediates for the introduction of reactive groups such as SiMe_3_ and I, and further functionalization [41]. In this frame, Erb et al. explored the possibility of obtaining enantioenriched derivatives **113** and **114** (Figure 21), determining their enantiomeric purity through HPLC analysis on Chiralpak IC‐3 (*n*‐hexane/2‐PrOH = 98:2) and IA‐3 (*n*‐hexane/2‐PrOH = 99:1), respectively. It is worth mentioning that the enantioseparation of the 2‐(trimethylsilyl)ferrocenecarboxaldehyde (**115**) was also performed on a cellulose‐based column (Chiralcel OD‐H column, *n*‐hexane/2‐PrOH 99:1 v/v) [186].

The enantioseparation of azaferrocenes was also reported by several groups. In 2001, Johannsen et al. reported the analytical enantioseparation of the sulfinyl azaferrocenes **56**, **116** and **117** (Figure S8) on the Chiralcel OD‐H (*n*‐hexane/2‐PrOH 90:10 v/v) [161]. The enantiomers *S*_s_,*S*_p_ and *R*_s_,*S*_p_ of the azaferrocene **116** were eluted at 12.1 and 10.9 min, respectively, whereas the enantiomers *R*_s_,*R*_p_ and *S*_s_,*S*_p_ of the other diastereoisomer remained unresolved under the adopted conditions. The enantiomers of compounds **117** (*t*_R_ = 11.1 min (*R*_p_) and 13.7 min (*S*_p_)) and **56** (*t*_R_ = 11.4 min (*S*_p_) and 20.1 min (*R*_p_)) were eluted with opposite elution order. In this case, it is interesting to note that the higher polarity of the distinctive substituent of **56** (R = CH_2_OH) compared to **117** (R = I) affects the elution of the second eluted enantiomer, whereas the first eluted enantiomers of the two azaferrocenes are eluted almost equally. Later, Iwao et al. also reported the HPLC analytical enantioseparation of azaferrocenes by using Chiralpak AD with *n*‐hexane/2‐PrOH 19:1 v/v as mobile phase [159].

### 3.2 Planar chiral ferrocenes containing aromatic groups and extended π‐clouds

**FIGURE 22:**
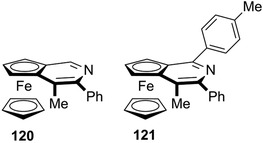
Structures of ferrocene‐fused pyridines **120** and **121**

As mentioned previously, in the late 1990s Fu's group reported the semipreparative enantioseparation of the ferrocene‐fused 4‐(dimethylamino)pyridine analogue **39** [28]. This derivative was one of the first planar chiral ferrocenes, containing π‐aromatic moieties, which were enantioseparated by enantioselective HPLC. Later, the same group reported the semipreparative enantioseparation of the 4‐(methyl)‐ (**118**) [187] and the 4‐(pyrrolidino)pyridine (**119**) [188] analogues on the Chiralcel OD by using *n*‐hexane/ethyl acetate/DEA 97:3:0.4 v/v/v and *n*‐hexane/ethanol/DEA 50:50:0.4 v/v/v as mobile phases, respectively. Very recently, Zheng et al. reported the enantioseparation of the two planar chiral ferrocene‐fused pyridines **120** and **121** (Figure 22) by using Chiralcel OD‐H (*n*‐hexane/2‐PrOH 95:5 v/v) and Chiralpak IG (*n*‐hexane/2‐PrOH 97:3 v/v), respectively, as chiral columns [142].

Starting from the 1980s [189], ferrocene helicenes, in which iron is included within the helical π‐framework, have been recognized as valuable systems for optoelectronic applications [190]. Indeed, coordination of the extended π‐electronic clouds to metal atoms impacts the electronic density distribution and, consequently, may change their properties. Given this context, in the last decade, enantioselective cycloisomerization reactions of 2‐ethynyl‐1‐ferrocenylbenzene derivatives affording helical‐shaped planar chiral ferrocenes have been investigated. In this context, the analytical enantioseparation of ferrocene‐fused polycyclic benzenes has been reported by several groups.

In 2016, Shibata et al. reported the analytical enantioseparation of nine planar chiral ferrocene‐fused polycyclic benzenes **122a**–**g**, **123** and **124** by using Chiralpak IA‐3, IB‐3 and IC‐3 as chiral columns under normal phase conditions (Table 4) [143]. From the chromatographic data summarized in Table 4, it emerges that enantioseparation of halogenated planar chiral ferrocenes, which do not contain polar groups, may be rather challenging, requiring the use of mobile phases with low elution strength. Urbano et al. also reported the analytical enantioseparation of compound **30** and of other analogues of the series **122** by using Chiralpak IA, IB and IC, as immobilized chiral columns, and in almost all cases *n*‐hexane/2‐PrOH mixtures, as mobile phases [31, 191]. Interestingly, the enantioseparations of **122e** and two other fluorinated analogues were performed on the Chiralpak IA under SFC conditions (CO_2_/MeOH 98:2) [191]. At the best of our knowledge, these represent the first examples of SFC enantioseparation of planar chiral ferrocenes reported in the literature.

**TABLE 4:** High‐performance liquid chromatography (HPLC) enantioseparation of planar chiral ferrocene‐fused polycyclic benzenes **122a**–**g**, **123** and **124** by using Chiralpak IA‐3, IB‐3 and IC‐3 as chiral columns under normal phase conditions [143]

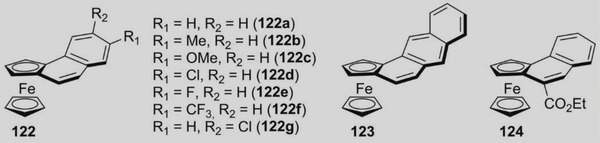				
Fc	Column	Mobile phase, *FR* (ml/min)^a^	*t*_R1_ (min)^b^	*t*_R2_ (min)^b^
**122a**	IB‐3	*n*‐Hexane/2‐PrOH 99:1, 0.5	14.6	17.1
**122b**	IC‐3	*n*‐Hexane/2‐PrOH 99:1, 0.5	15.9	18.9
**122c**	IB‐3	*n*‐Hexane/2‐PrOH 99:1, 0.5	18.3	21.0
**122d**	IA‐3	*n*‐Hexane/2‐PrOH 99.5:0.5, 0.5	20.1	21.6
**122e**	IA‐3	*n*‐Hexane/2‐PrOH 99.5:0.5, 0.5	20.1	21.9
**122f**	IA‐3	*n*‐Hexane/2‐PrOH 99.5:0.5, 0.5	19.2	20.2
**122g**	IA‐3	*n*‐Hexane/2‐PrOH 99.5:0.5, 0.5	19.2	25.2
**123**	IC‐3	*n*‐Hexane/2‐PrOH 99:1, 1.0	14.3	17.8
**124**	IC‐3	*n*‐Hexane/2‐PrOH 95:5, 1.0	7.9	14.2

^a^ Flow rate.

^b^ Retention time.

**FIGURE 23:**
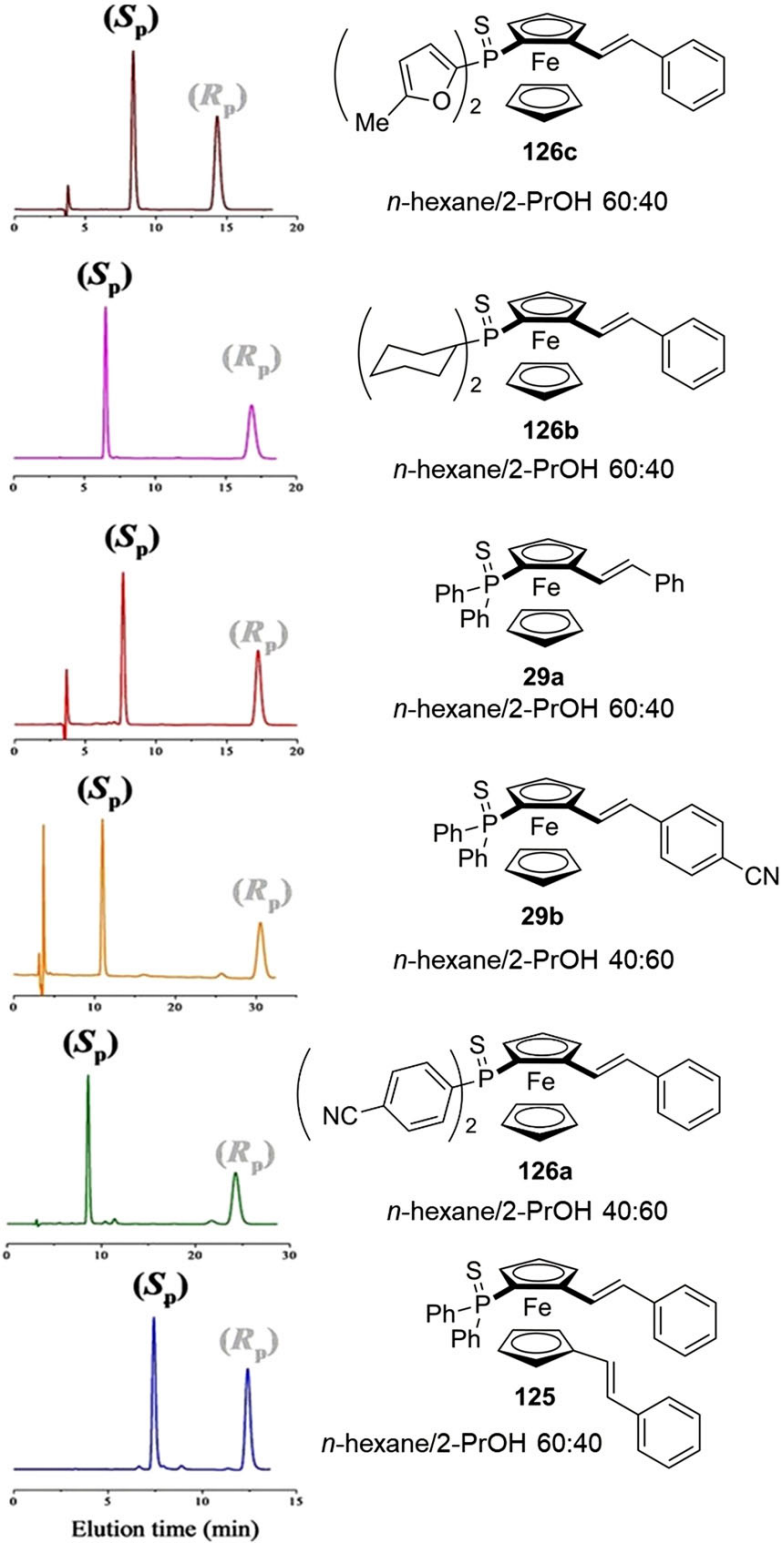
High‐performance liquid chromatography (HPLC) enantioseparation of planar chiral ferrocenes **29a**,**b**, **125** and **126a**–**c** on Chiralpak IG. *Source*: Adapted from Ref. [34] with permission

In 2020, Lang et al. reported the successful discrimination of the enantiomers of planar chiral ferrocenes **29a**,**b**, **125** and **126a**–**c** (Figure 23) in voltammetry experiments on electrodes modified with electrodeposited inherently chiral oligomer films [34]. In this context, the chromatographic enantioseparations of these ferrocenes were carried out using the Chiralpak IG‐3, as chiral column, with normal phase eluents. As shown in Figure 23, excellent enantiomer separation was achieved in all cases, and the process was scaled up to a semipreparative level, where both enantiomers could be collected in multi‐milligram quantities.

**FIGURE 24:**
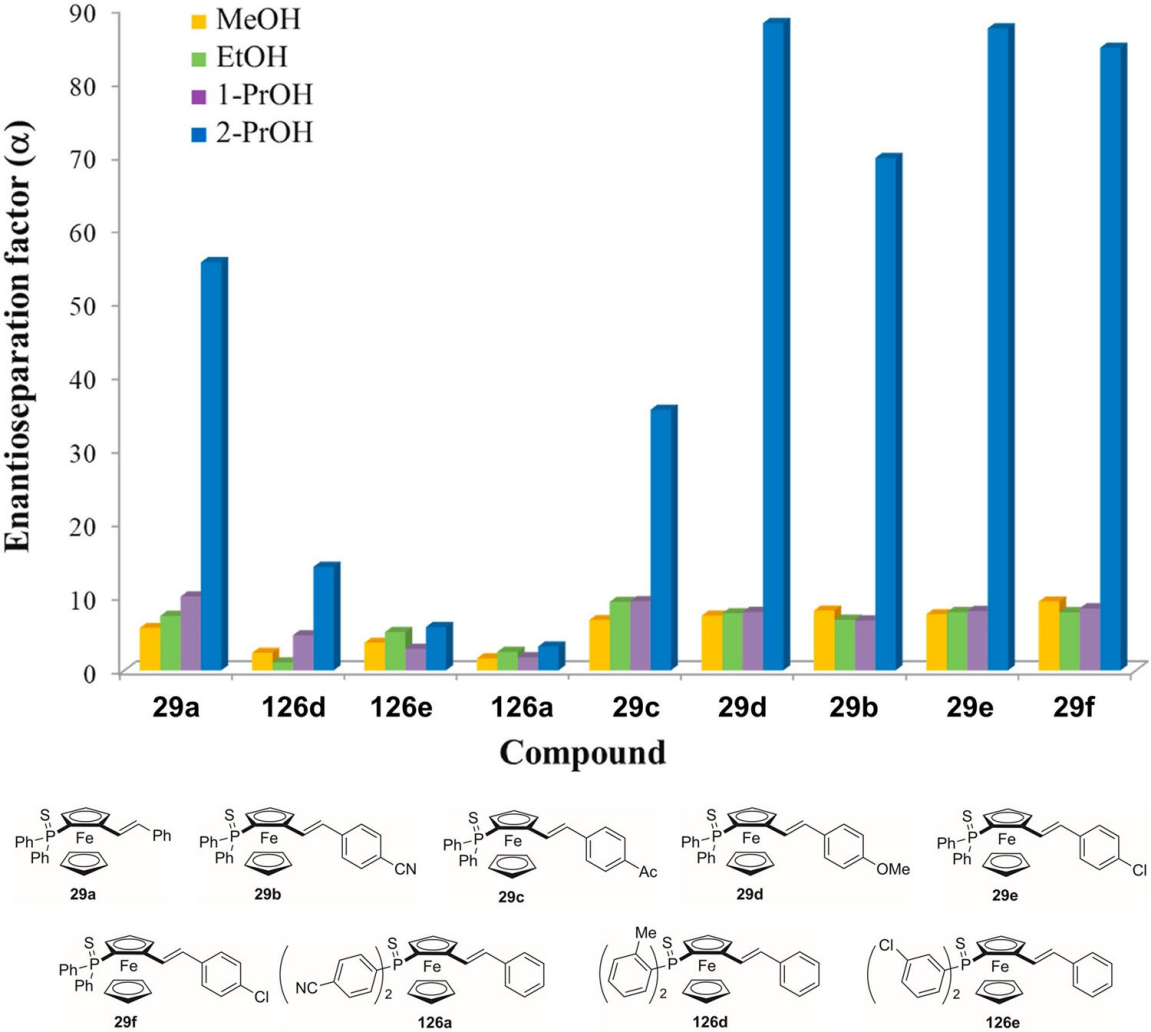
Effect of mobile phase on the enantioselectivity of ferrocenes **29a**–**f** and **126a**,**d**,**e** on Chiralpak AD‐3. *Source*: Adapted from Ref. [47] with permission

Very recently, Cirilli et al. investigated the HPLC enantioseparation of the two series of planar chiral ferrocenes **29b**–**f** and **126a**,**d**,**e** on the Chiralpak AD‐3, as chiral column (Figure 24) [47], considering compound **29a** as reference for comparison. Although the first series **29** carried different substituents on the styryl moiety, compounds **126** presented distinctive substituents on the thiophosphoryl moiety. The enantioseparations were carried out using pure MeOH, EtOH, 1‐PrOH and 2‐PrOH as well as mixtures of *n*‐hexane/2‐PrOH as mobile phases. Interestingly, in this study, it was found that the enantioseparation extent was significantly influenced by elution modes and the steric hindrance of substituted aromatic rings. As shown in Figure 24, exceptional selectivity factors could be observed in certain conditions. For the unsubstituted **29a**, the enantioselectivity increased as the mobile phase changed from MeOH to 1‐PrOH, ranging from 5.76 to 10.04. By using 2‐PrOH, selectivity factor increased up to 55.51. For ferrocenes **126**, selectivity was less influenced by the nature of alcoholic eluent, the highest selectivity factor observed for **126d** with 2‐PrOH as mobile phase (*α* = 14.07). On this basis, the presence of substituents on the phenyl‐thiophosphoryl fragment appeared to be detrimental for enantioseparation. For the series **29b**–**f**, the most remarkable difference in enantioselectivity was obtained with 2‐PrOH, and the highest selectivity values were observed for compounds **29d** (*α* = 88.05), **29e** (*α* = 87.33) and **29f** (*α* = 84.68). For all this type of exceptional enantioseparations, the EEO was *S*_p_–*R*_p_.

**FIGURE 25:**
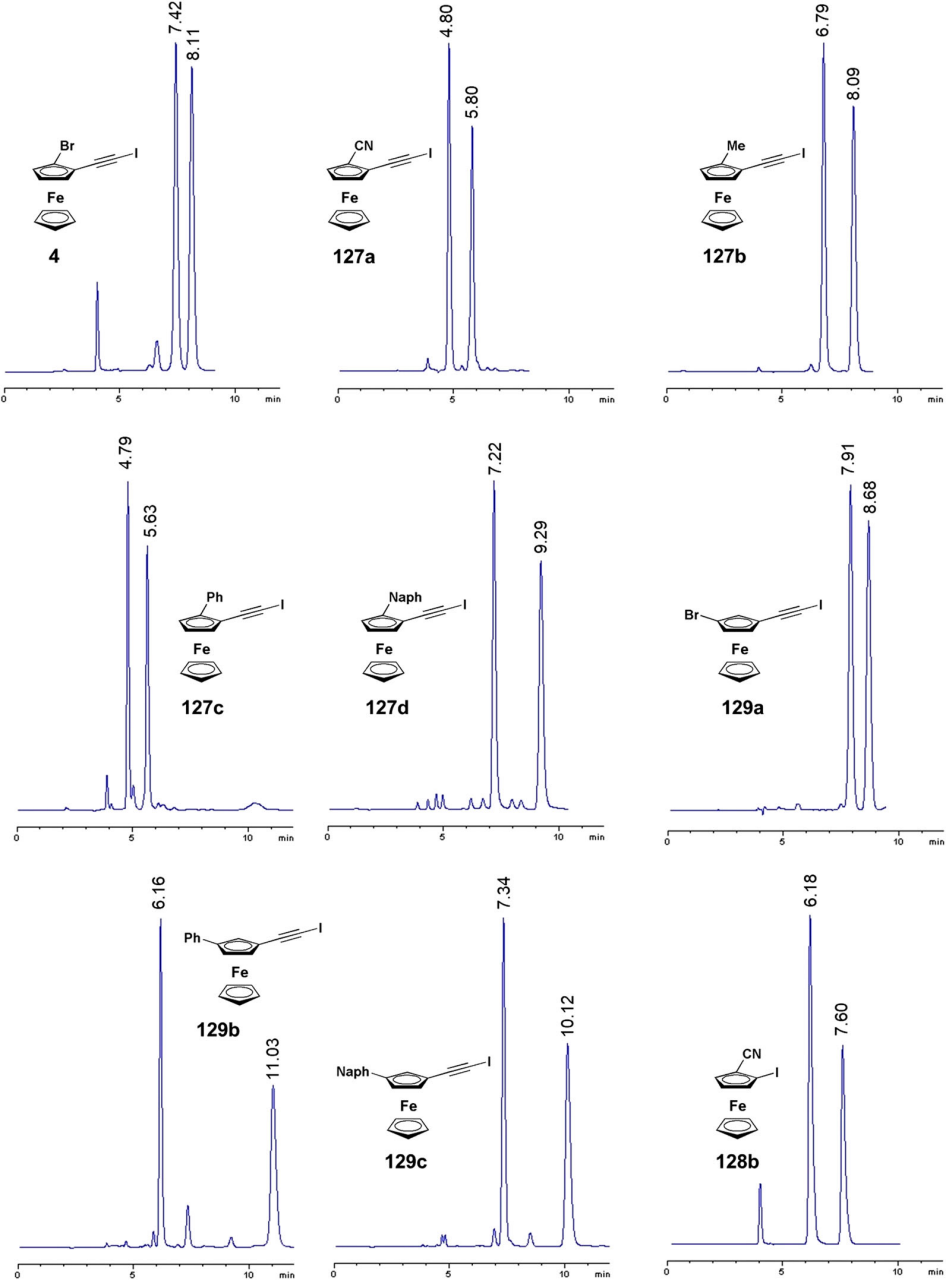
Baseline enantioseparation traces of compounds **4**, **127a**–**d**, **128b** and **129a**–**c**. Chromatographic conditions: **4**, Lux Amylose 1, MeOH/water 90:10; **127a**, Lux Amylose 1, MeOH/water 95:5; **127b**, Lux i‐Amylose 3, MeOH/water 90:10; **127c**, Lux i‐Amylose 3, MeOH 100%; **127d**, Lux i‐Cellulose 5, *n*‐hexane/2‐PrOH 95:5; **129a**, Lux Amylose‐1, MeOH 100%; **129b** and **129c**, Lux i‐Amylose 3, *n*‐hexane/2‐PrOH 95:5; **128b**, Lux Cellulose‐1, MeOH/water 95:5. *Source*: Adapted from Ref. [45] with permission

**FIGURE 26:**
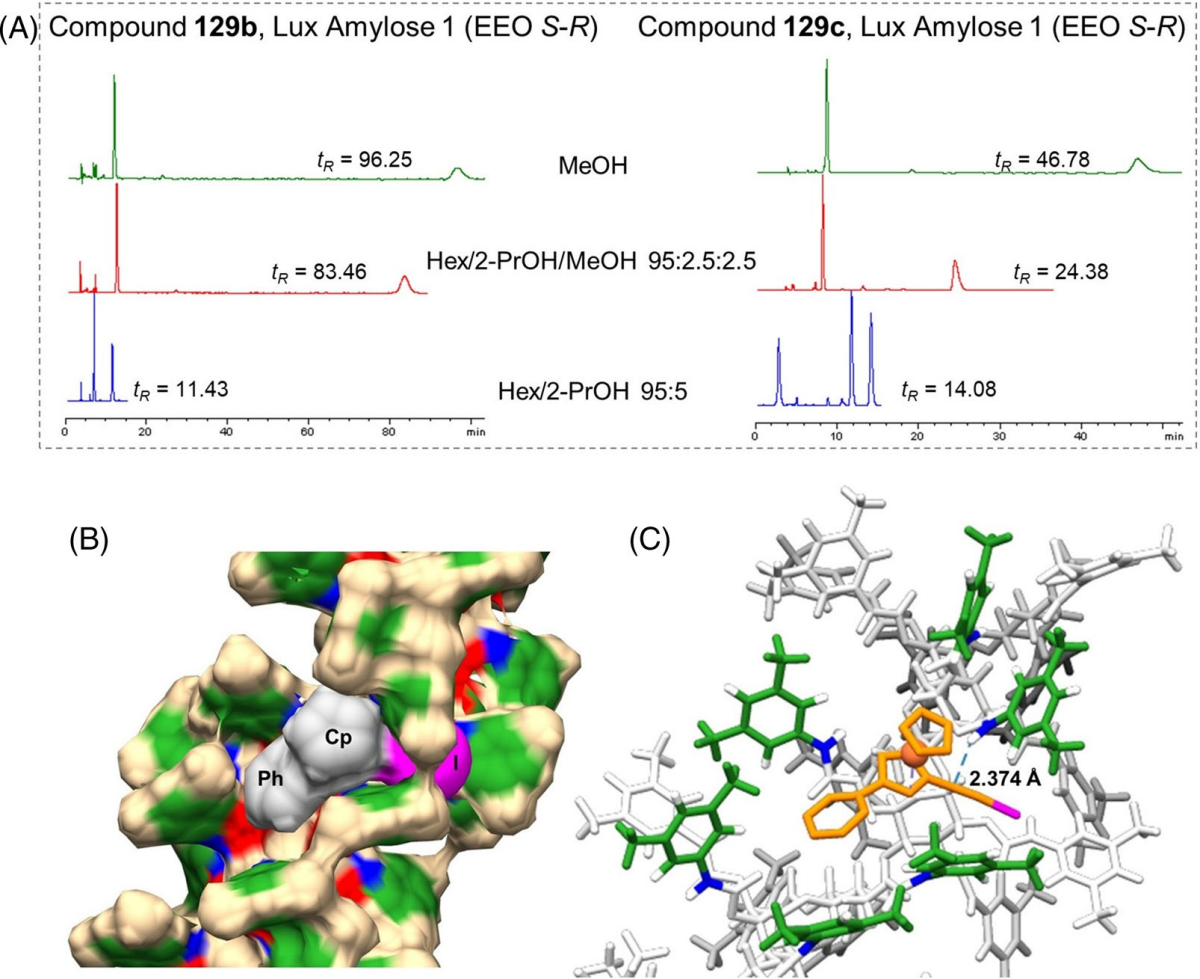
Comparison of the enantioseparation traces of compounds **129b** and **129c** on Lux Amylose 1, under multimodal elution conditions (A). Representative snapshot from the simulated MD trajectories (100 ns) of (*R*_p_)–**129b** complex with amylose tris(3,5‐dimethylphenylcarbamate) (solvent box, MeOH): (B) electron density surface (legend colors: green, aromatic ring; red, C=O; blue, N–H; gray, Ph + Cp + C≡C of (*R*_p_)–**129b**; magenta, iodine) and (C) tube model of the (*R*_p_)–**129b**/selector complex (legend colors: orange, Ph + Cp + Fe + C≡C of (*R*_p_)–**129b**; magenta, iodine; green, aromatic rings featuring and delimiting the hydrophobic binding cavity of the selector). *Source*: Adapted from Ref. [45] with permission

As reported earlier, moderate/high enantioselectivities can be obtained for planar chiral ferrocenes bearing polar substituents, whereas the enantioseparations of derivatives containing halogens, or exclusively alkyl groups, often proved to be rather challenging. In this regard, recently our groups explored the enantioseparation of 10 planar chiral 1,2‐ (**4**, **127a**–**d** and **128a,b**) and 1,3‐disubstituted (**129a**–**c**) ferrocenes by using five polysaccharide‐based CSPs (Lux Cellulose‐1, i‐Cellulose‐5, Amylose‐1, i‐Amylose‐1 and i‐Amylose‐3) under multimodal elution conditions [45]. In this study, baseline enantioseparations were achieved for nine analytes with *α* ranging from 1.20 to 2.92 (Figure 25). Otherwise, 1‐bromo‐2‐iodo‐ferrocene (**128a**) could be only partially enantioseparated, confirming that the enantioseparation of small nonpolar planar chiral ferrocenes may be not easy due to the inherent structural inability of the enantiomers of this type of molecular systems to be enantiodifferentiated. In most cases, amylose‐based CSPs provided better enantioseparation compared to cellulose‐based ones. Moreover, due to the hydrophobic feature of the ferrocenes used in the study as analytes, aqueous methanol‐containing MPs allowed for improving enantioseparation performances in several cases. Interestingly, for compound **129b**, the presence of π‐extended systems in the analyte structure was shown to impact affinity of the most retained enantiomer towards amylose‐based selectors, observing very high selectivity factor (*α* = 11.41) with pure MeOH as mobile phase, with *S*_p_–*R*_p_ as EEO (Figure 26A). In particular, it was observed that the addition of small amounts of MeOH to an *n*‐hexane/2‐PrOH mixture increased significantly the affinity of the second eluted enantiomers of both compounds **129b** and **129c**, producing large enantioseparation on amylose‐based selectors. With the aim to disclose the origin of this phenomenon at molecular level, molecular dynamic (MD) simulations were performed by using the *R*_p_‐enantiomer of **129b** and a virtual model of the amylose tris(3,5‐dimethylphenylcarbamate) (Figure 26B,C). This computational analysis confirmed (a) the confinement of the analyte in a hydrophobic cavity deeply inside the selector groove and (b) the stabilization of the selector–selectand complex through an HB (*d* = 2.347 Å) between the ethynyl π‐cloud and the amidic hydrogen of the selector as the molecular basis of the observed large enantioseparation.

In the last few years, several efforts have also been made to prepare enantiomerically enriched ferrocenes featured by π‐electronic clouds. Indeed, the development of advanced computational methods has allowed to recognize that catalysts equipped with large aromatic groups may provide cumulative noncovalent interactions such as C–H⋯π, N–H⋯π and π⋯π with a remarkable stabilization force on the catalytic transition states, often with the participation of dispersion forces [192]. In this frame, in the last few years, the analytical enantioseparation of substituted 4‐methyl‐6‐(methylsulfonyl)‐6*H*‐benzo[2,3]‐azepino[4,5‐*a*]ferrocenes **35a**–**g** (Table 5) [144], of 1‐*N*,*N*‐diisopropyl‐2‐substituted‐ferroceneamides **130** (Figure S9) [193] and of 1‐[(2*E*)‐(4‐substituted‐benzylidene)hydrazono]methyl]‐2‐substituted‐ferrocenes **38** (Figure S9) [147] were reported. As shown in Table 5, for the enantioseparation of planar chiral ferrocenes containing extended π‐electronic cloud regions, methylated polysaccharide‐based selectors, in particular amylose‐based ones, are preferred along with mobile phases featured by low eluting strength. However, under these conditions, phenomena of peak broadening and low resolution may be observed. Accordingly, for the analytical enantioseparation of 23 ferrocenes of the series **130**, in most cases chiral columns based on methylated polysaccharide selectors such a Chiralcel OD‐3 and Chiralpak AD‐H, and *n*‐hexane/2‐PrOH mixtures with 2‐PrOH ≤2% were used [193]. Analogously, in the case of 22 compounds of the series **38**, methylated Chiralpack AD‐H and Chiralcel OD‐H were used as chiral columns, exclusively [147].

**TABLE 5:** High‐performance liquid chromatography (HPLC) enantioseparation of planar chiral substituted 4‐methyl‐6‐(methylsulfonyl)‐6*H*‐benzo[2,3]‐azepino[4,5‐*a*]ferrocenes **35a**–**g** by using Chiralpak IA and IB‐3 under normal phase conditions [193]

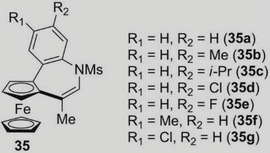				
Fc	Column	Mobile phase, *FR* (ml/min)^a^	*t*_R1_ (min)^b^	*t*_R2_ (min)^b^
**35a**	IB‐3	*n*‐Hexane/2‐PrOH 97:3, 1.0	20.0	21.6
**35b**	IA	*n*‐Hexane/2‐PrOH 97:3, 1.0	15.0	16.7
**35c**	IA	*n*‐Hexane/2‐PrOH 97:3, 1.0	12.0	12.8
**35d**	IA	*n*‐Hexane/2‐PrOH 97:3, 1.0	26.3	28.9
**35e**	IA	*n*‐Hexane/2‐PrOH 97:3, 1.0	17.2	19.2
**35f**	IA	*n*‐Hexane/2‐PrOH 99:1, 1.0	32.7	35.1
**35g**	IA	*n*‐Hexane/2‐PrOH 99:1, 1.0	32.8	35.7

^a^ Flow rate.

^b^ Retention time.

### 3.3 Planar chiral ferrocenes containing halogens as substituents

Halogenated ferrocenes are key intermediates for accessing polysubstituted ferrocenes [194]. More recently, iodinated ferrocenes were found to be involved in XB in solid state [106, 195], and even in solution with promising applications as organocatalysts in organic synthesis [87, 106].

In 2020, Kumar et al. described the analytical enantioseparation of the *N*,*N*‐diisopropyl‐2‐iodoferroceneamide (**131**) on Chiralcel OD‐3 with *n*‐hexane/2‐PrOH 98:2 v/v as mobile phase [193]. Later, Erb et al. reported the analytical enantioseparation of the series of iodinated planar chiral ferrocenes **132a**–**e** by using Chiralpak IA‐3 and IC‐3 as chiral columns under normal phase conditions (Table 6) [196]. It is worth noting that, given the hydrophobic character of iodine, the enantioseparation of some members of the series required the use of *n*‐hexane‐based mobile phases containing just 1% 2‐PrOH.

**TABLE 6:** High‐performance liquid chromatography (HPLC) enantioseparation of iodinated planar chiral ferrocene **132a**–**e** with Chiralpak IA‐3 and IC‐3 under normal phase conditions [196]

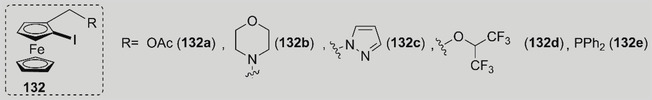					
Fc	Column	Mobile phase, *FR* (ml/min)^a^	*t*_R1_ (min)^b^	*t*_R2_ (min)^b^	EEO^c^
**132a**	IC‐3	*n*‐Hexane/2‐PrOH 99:1, 0.9	16.8	17.4	*S*_p_–*R*_p_
**132b**	IA‐3	*n*‐Hexane/2‐PrOH 95:5, 1.0	7.0	7.3	*S*_p_–*R*_p_
**132c**	IC‐3	*n*‐Hexane/2‐PrOH 90:10, 0.8	11.9	13.4	*S*_p_–*R*_p_
**132d**	IA‐3	*n*‐Hexane/2‐PrOH 99:1, 0.5	11.0	11.7	*R*_p_–*S*_p_
**132e**	IA‐3	*n*‐Hexane/2‐PrOH 99:1, 0.8	6.2	6.8	*S*_p_–*R*_p_

^a^ Flow rate.

^b^ Retention time.

^c^ Enantiomer elution order.

**FIGURE 27:**
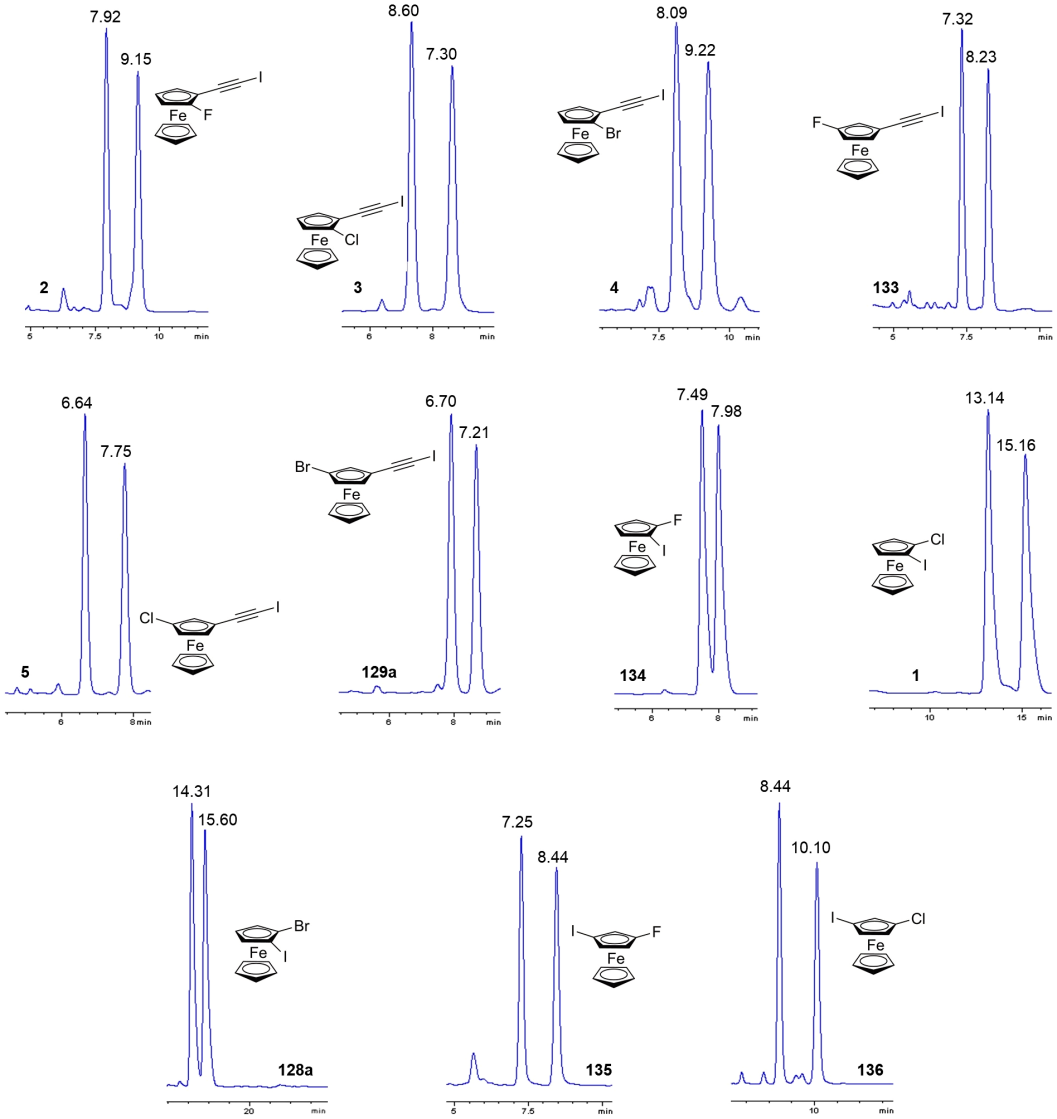
Baseline enantioseparation traces of compounds **1**–**5**, **128a**, **129a** and **133**–**136**. Chromatographic conditions (*T* = 25°C if not indicated otherwise): **2** and **3**, Lux Amylose‐1, MeOH/water 90:10 v/v; **4**, Lux Amylose‐1, MeOH/water 90:10 v/v (*T* = 10°C); **133**, Lux i‐Amylose‐1, MeOH/water 95:5 v/v; **5** and **129a**, Lux Amylose‐1, MeOH 100%; **134**, Lux Cellulose‐1, *n*‐hexane/2‐PrOH 95:5 v/v (*T* = 5°C); **1** and **128a**, Lux Cellulose‐1, MeOH/water 90:10 v/v (*T* = 5°C); **135** and **136**, Lux Amylose‐1, MeOH/water 95:5 v/v. *Source*: Adapted from Ref. [46] with permission

**FIGURE 28:**
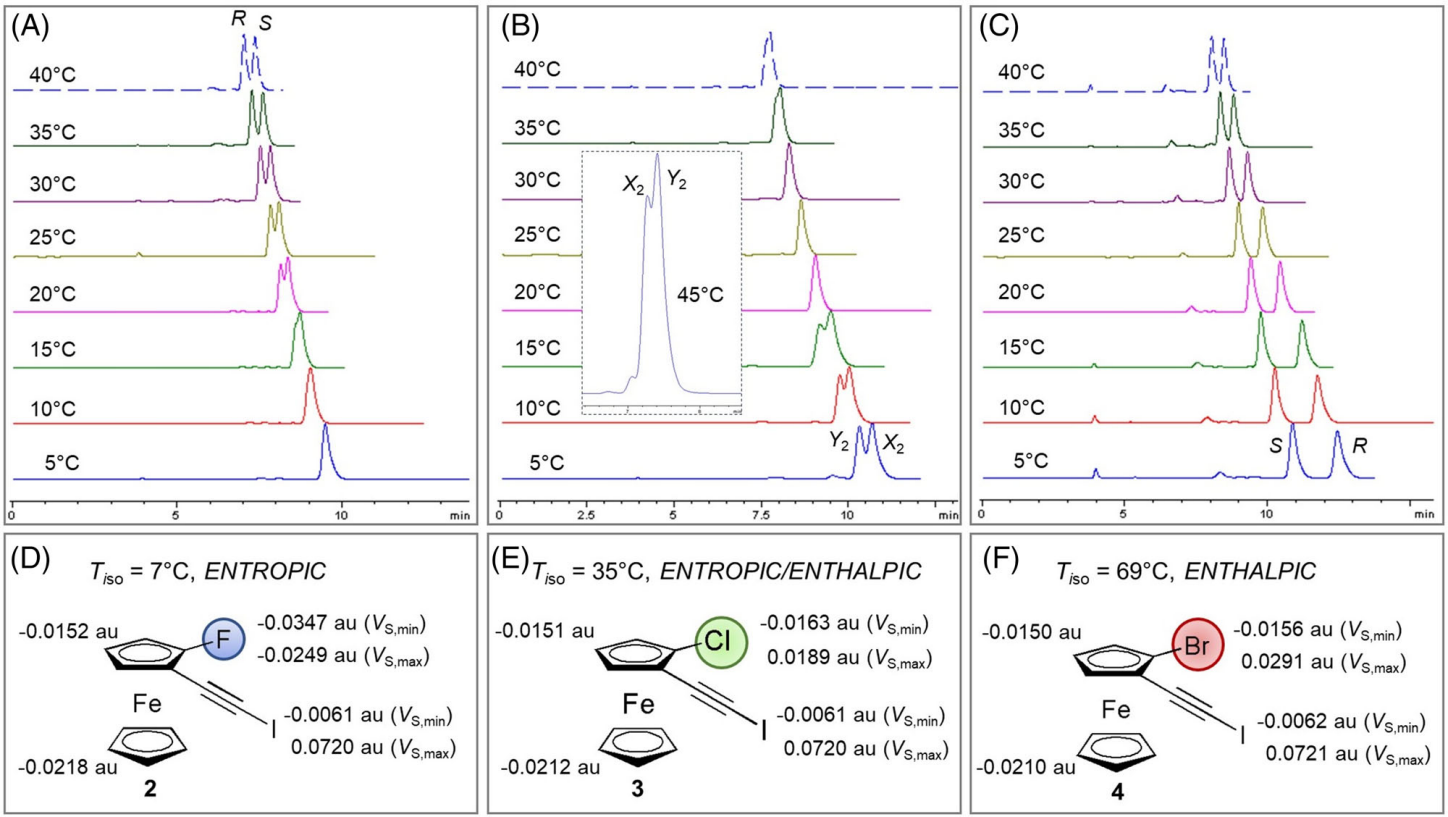
Enantioseparation of ferrocenes **2** (A), **3** (B) and **4** (C) at variable temperature on Lux Cellulose‐1 with *n*‐hexane/2‐PrOH 95:5 v/v, and variation of the *V*_S,min_ and *V*_S,max_ values (D–F) as the 1‐halogen substituent changes in the series of 1‐halo‐2‐(iodoethynyl)ferrocenes **2**–**4**. *Source*: Adapted from Ref. [46] with permission

Very recently, our groups explored systematically the enantioseparation of 11 dihalogenated planar chiral ferrocenes **1**–**5**, **128a**, **129a** and **133**–**136** by using 5 polysaccharide‐based CSPs (Lux Cellulose‐1, i‐Cellulose‐5, Amylose‐1, i‐Amylose‐1 and i‐Amylose‐3) under multimodal elution conditions [46]. Baseline enantioseparations were achieved for nine analytes with *α* ranging from 1.15 to 1.66 (Figure 27). In this study, the impact of CSP structure and mobile phase polarity on the enantioseparation was evaluated. In all cases, the best selectivity values were obtained by using Lux Amylose‐1 and i‐Amylose‐3 as CSPs with methanol‐containing mobile phases, with the exception of 1‐bromo‐2‐iodo‐ferrocene **128a** (*α* = 1.13) and 1‐fluoro‐2‐iodo‐ferrocene **134** (*α* = 1.12) which showed better enantioseparation on Lux Cellulose‐1. Moreover, the impact of halogen type and position on the enantioseparation was investigated by correlating theoretical and experimental data. For this purpose, thermodynamic quantities associated with the enantioseparations were derived from van 't Hoff plots, and for 1‐halo‐2‐(iodoethynyl)ferrocenes (1‐halogen = F, Cl, Br) **2**–**4**, halogen‐dependent thermodynamic profiles were identified on the cellulose tris(3,5‐dimethylphenylcarbamate)‐based column (Figure 28). In particular, with the aim to unravel the functions of halogen substituents in mechanisms and noncovalent interactions underlying selector‐selectand complex formation at molecular level, local electron charge density of specific molecular regions of the interacting partners were evaluated in terms of calculated *V* and related source function contributions. On this basis, it was demonstrated that HB involving the NH group of the selector as the HB donor participates in the enantiodifferentiation mechanism for the fluorinated ferrocene **2**, whereas an XB involving the carbonyl group of the selector occurred for the more polarizable 1‐bromoferrocene **4**.

**FIGURE 29:**
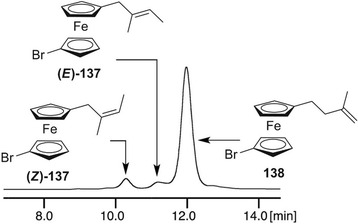
High‐performance liquid chromatography (HPLC) traces for the mixture of (*E*)‐ and (*Z*)–**137** and **138** on Chiralpak IA using *n*‐hexane as an eluent

It is worth mentioning that, recently, Ohnishi et al. used Chiralpak IA, IB and IC, for the separation of nonenantiomeric isomers of various ferrocenes. In particular, as shown in Figure 29, the (*E*)‐ and (*Z*)‐isomers of 1‐bromo‐1′‐(2‐methyl‐2‐butenyl)ferrocene **137** and 1‐bromo‐1′‐(3‐methyl‐3‐butenyl)ferrocene **138** could be separated by using Chiralpak IA as column and pure *n*‐hexane as mobile phase (*FR* = 0.5 ml/min) [197].

## 4 CONCLUDING REMARKS

Chiral planar ferrocenes have been attracting a huge interest in several fields from asymmetric catalysis to medicinal chemistry, electrochemistry and chiroptical analysis. Despite that, the access to enantiopure or enantioenriched planar chiral ferrocenes on a wide scale is limited by the fact that a number of motifs still lack efficient asymmetric strategies. In this regard, enantioseparation science may definitely contribute to fill this gap. However, the overview of the studies developed over time suggests that these issues have been rather overlooked by analytical scientists. As a consequence, a huge number of enantioseparations of planar chiral ferrocenes have been carried out in the field of organic and organometallic asymmetric synthesis with the purpose to determine the enantiomeric excesses of chiral products, whereas few systematic analytical studies have been reported over time. Nowadays, electromigration techniques remain almost unexplored in this field, and only very recently the first CE enantioseparations of planar chiral ferrocenes were reported by the Lipka's group by using native and substituted CDs as chiral selectors [198].

Although the first enantioseparations of a planar chiral ferrocene were performed on CD‐based chiral columns, and some enantioseparations by using the Whelk‐O1 column have also been reported, most analyses in this field have been performed by using polysaccharide‐based CSPs. Due to the high load capacity of these CSPs, starting from the late 1990s, planar chiral ferrocene catalysts were available in their enantiopure form through preparative HPLC enantioseparation.

With the aim to obtain baseline enantioseparation (*R*_s_ ≥ 1.5) of planar chiral ferrocenes, focused choices have to be done, in terms of chiral column and elution mode, which are strictly dependent on structure and properties of the substituents generating the chiral plane. In 1,2,*n*′‐ and 1,3,*n*′‐trisubstituted planar chiral ferrocenes, the substituents at the *n*′ position may impact retention and selectivity depending on their polarity and steric hindrance. On the basis of the results available so far, an attempt to profile some practical guidelines for approaching the enantioseparation of planar chiral ferrocenes with polysaccharide‐based selectors can be made: (a) chlorinated polysaccharide‐based ones were successfully used for the enantioseparation of polar ferrocenes containing carbonyl groups and carboxamide moieties; (b) otherwise, methylated polysaccharide‐based CSPs showed better performances with nonpolar analytes. In these cases, methylated and chloromethylated amylose‐based columns exhibit better performances compared to cellulose‐based columns; (c) in general, ferrocenes containing nonpolar and hydrophobic groups may be challenging to enantioseparate under normal phase conditions. In this case, the elution strength of *n*‐hexane‐based mobile phases needs to be reduced by using percentages of 2‐PrOH ≤5%. Alternatively, pure *n*‐hexane or *n*‐heptane may be used as mobile phase, although with these solvents peak broadening is not unusual, and the analytes may be poorly soluble in the mobile phase. In these cases, methanol‐containing mixtures may provide better results in terms of selectivity factors compared to classical *n*‐hexane/2‐PrOH mixtures.

Recent studies have paved the way to new horizons, generating a renewed interest in the enantioseparation of planar chiral ferrocenes. On this basis, the new trends in this field are (a) *methods development and applications under SFC conditions*, which may open new possibilities for preparative enantioseparation of planar chiral ferrocenes. So far, just two groups performed SFC enantioseparations of planar chiral ferrocenes in the frame of organic synthesis studies [124, 191], and only recently the first analytical study under SFC conditions has been published [48]; (b) *methods based on the use of polar organic mode and aqueous–organic mixtures* which have been proven to be more efficient for the enantioseparation of nonpolar and hydrophobic ferrocene compared to the popular *n*‐hexane/2‐PrOH mixtures [45, 46]; (c) *mechanistic studies on high and exceptionally high enantioseparations* provided by planar chiral ferrocenes featuring aromatic groups [45, 47]. This type of phenomena seems to be strictly dependent on polar solvents such as MeOH or 2‐PrOH, proving that mobile phase, depending on its properties (polarity) may switch on or switch off some specific noncovalent interactions underlying binding and recognition; (d) *ferrocenes containing extended π‐electronic clouds* may function as privileged test probes to explore π–π and dispersion forces in HPLC environment; (e) *halogenated ferrocenes containing electrophilically activated iodine substituents* were shown to participate in XBs under normal phase conditions; thus, halogenated ferrocenes may be used as test probes to explore properties and function of XBs in HPLC enantioseparation [46].

Despite the fact that integrating experimental and computational analysis represents a powerful tool to unravel the bases of enantioseparation mechanisms at the molecular level, modelling of ferrocene enantioseparation is still in its infancy. The first approaches to explore the enantioseparation of planar chiral ferrocenes by using computational techniques were developed only recently by our groups by using MD simulations, *V* analysis and related source function reconstruction [45, 46].

## CONFLICT OF INTEREST

The authors have declared no conflict of interest.

## Supporting information

Supporting Information

## Data Availability

The data that support the findings of this study are available from the corresponding author upon reasonable request.
