# Supplementary material for: Ferrocene derivatives with planar chirality and their enantioseparation by liquid‐phase techniques
Source: Electrophoresis. 2022 Sep 4;44(1-2):158–89. doi: 10.1002/elps.202200148 (PMC10087518; doi:10.1002/elps.202200148)
Supplement: Supplementary file 1 — Supporting Information [file ELPS-44-158-s001.pdf]

Supplementary materials

Ferrocene derivatives with planar chirality and their enantioseparation by liquid-phase techniques

Paola Peluso,[1] and Victor Mamane[2]

[1] Istituto di Chimica Biomolecolare ICB CNR, Sede secondaria di Sassari, Sassari, Italy.
[2] Institut de Chimie de Strasbourg, UMR 7177, CNRS-Université de Strasbourg, Strasbourg, France.

*Correspondence should be addressed to the following authors:

Dr. Paola Peluso
Istituto di Chimica Biomolecolare, Consiglio Nazionale delle Ricerche
Traversa La Crucca 3, Li Punti, 07100 Sassari, Italy, paola.peluso@cnr.it

Dr. Victor Mamane
Institut de Chimie de Strasbourg, Centre National de la Recherche Scientifique and Université de Strasbourg
1 rue Blaise Pascal, 67008 Strasbourg Cedex, France, vmamane@unistra.fr

Table of contents pag.

**1**	**Introduction**	
**1.1**	**General features of the ferrocene structure: additional materials**	
	Figure S1	2
**1.2**	**Basic concepts of ferrocene modelling: additional materials**	
	Table S1	2
**2**	**Planar chiral ferrocenes**	
**2.**	**Recent applications of planar chiral ferrocenes: additional materials**	
	Figure S2	3
	Figure S3	3
	Figure S4	3
	Figure S5	4
	Figure S6	4
**3**	**Enantioseparation of planar chiral ferrocenes: additional materials**	
	Figure S7	5
	Table S2	5
**3.1**	**Planar chiral ferrocenes containing polar groups: additional materials**	
	Table S3	6
	Table S4	7
	Table S5	8
	Table S6	9
	Table S7	10
	Table S8	12
	Figure S8	13
**3.2**	**Planar chiral ferrocenes containing aromatic groups: additional materials**	
	Figure S9	13

1 Introduction

1.1 General features of the ferrocene structure: additional materials

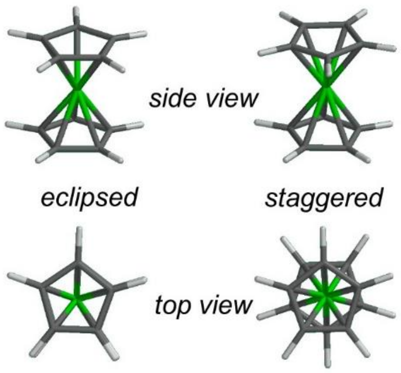

Figure S1. Tube models of the eclipsed and staggered conformations of ferrocene (colour legend: green, iron; grey, carbon; pale grey, hydrogen).

1.2 Basic concepts of ferrocene modelling: additional materials

Table S1. Magnitude of iodine σ-holes (kJ/mol) calculated at DFT level of theory with the B3LYP-D3 and B3LYP functionals and the def2-TZVPP as basis set for ferrocenes **1-5** a)

Fc	B3LYP-D3	B3LYP
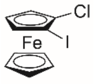 **1**	119 (1.00)	122 (1.00)
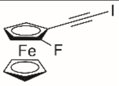 **2**	182 (1.53)	189 (1.55)
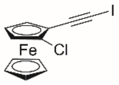 **3**	182 (1.53)	189 (1.55)
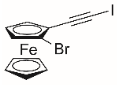 **4**	182 (1.53)	189 (1.55)
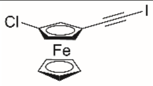 **5**	185 (1.55)	193 (1.58)

a) The relative magnitudes normalized with respect to the ferrocene analogue **1** are provided in parentheses

2 Planar chiral ferrocenes

2.1 Recent applications of planar chiral ferrocenes: additional materials

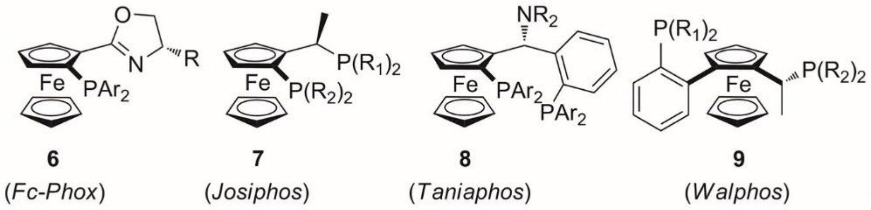

Figure S2. Structures of well-known P,N- and P,P-ferrocene ligands.

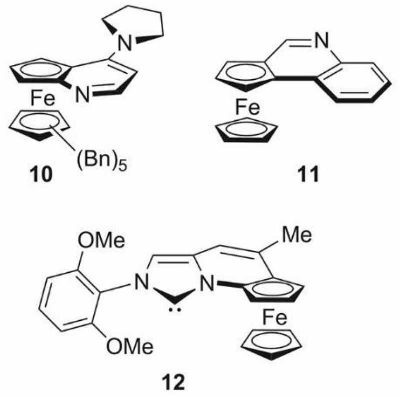

Figure S3. Recently reported ferrocene-fused nitrogen heterocyclic organocatalysts **10**-**12**.

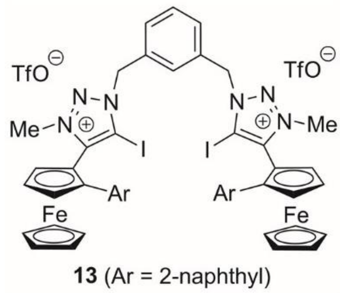

Figure S4. Structure of planar chiral bis-triazolium ferrocenyl catalyst **13**.

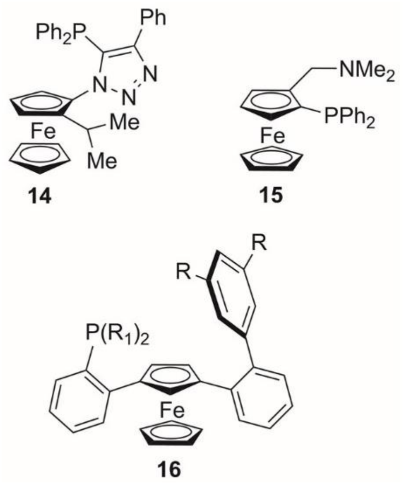

Figure S5. Ferrocenyl phosphines **14-16** with planar chirality.

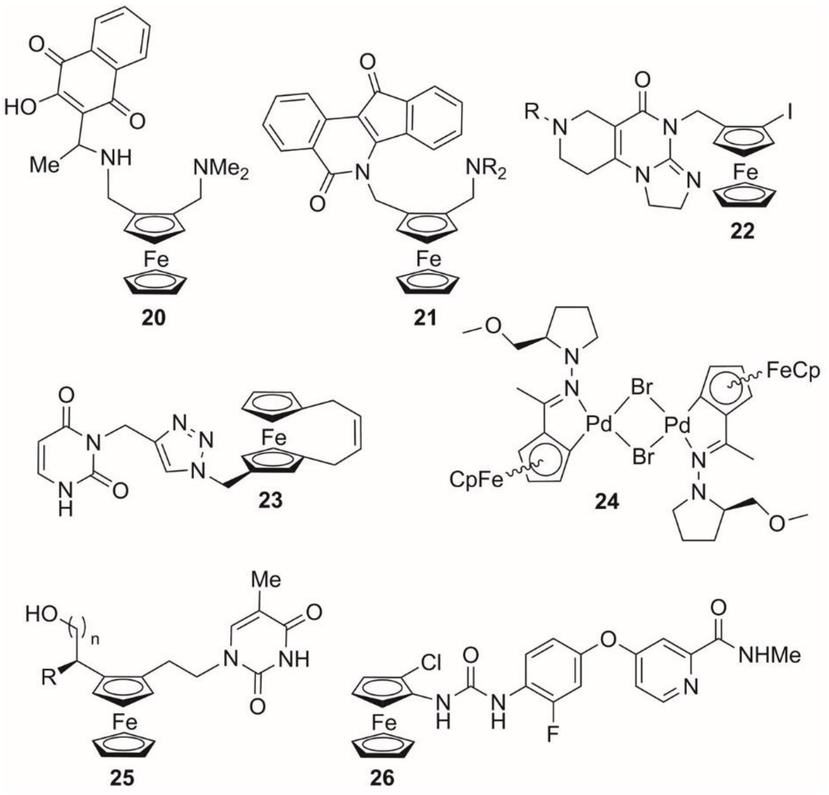

Figure S6. Planar chiral ferrocenes with biological activity.

3 Enantioseparation of planar chiral ferrocenes: additional materials

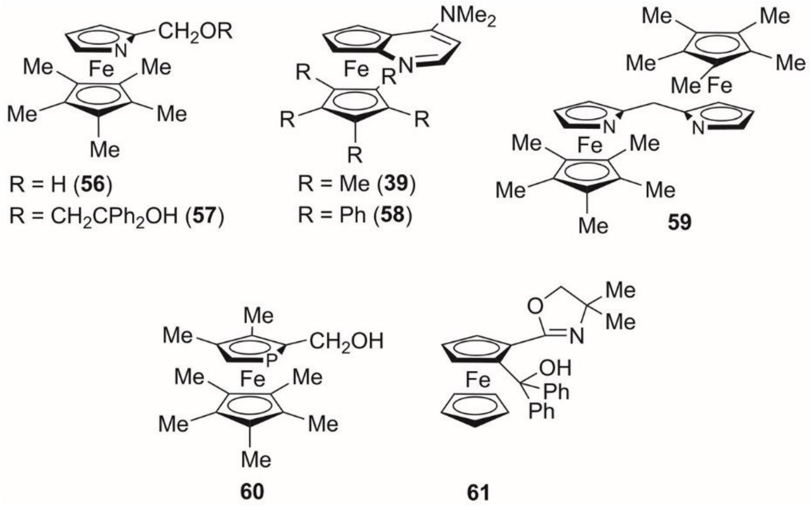

Figure S7. First planar chiral ferrocenes enantioseparated at semi-preparative level by HPLC.

Table S2. Distinctive structures of chiral columns based on derivatized polysaccharides [a)]

column name	type [b)]	chiral selector
Chiralpak AD (AD-H)	coated	amylose *tris*(3,5-dimethylphenylcarbamate)
Lux Amylose-1	coated	
Chiralpak IA	immobilized	
Lux i-Amyose-1	immobilized	
Chiralpak IE	immobilized	amylose *tris*(3,5-dichlorophenylcarbamate)
Chiralpak IF	immobilized	amylose *tris*(3-chloro-4-methylphenylcarbamate)
Chiralpak IG	immobilized	amylose *tris*(3-chloro-5-methylphenylcarbamate)
Lux i-Amylose-3	immobilized	
Chiralpak AY	coated	amylose *tris*(5-chloro-2-methylphenylcarbamate)
Lux Amylose-2	coated	
Chiralpak AS-H	coated	amylose *tris*((*S*)-α-methylbenzylcarbamate)
Chiralcel OD (OD-H)	coated	cellulose *tris*(3,5-dimethylphenylcarbamate)
Lux Celllulose-1	coated	
Chiralpak IB	immobilized	
Chiralpak IC	immobilized	cellulose *tris*(3,5-dichlorophenylcarbamate)
Lux i-Cellulose-5	immobilized	
Chiralcel OZ	coated	cellulose *tris*(3-chloro-4-methylphenylcarbamate)
Lux Cellulose-2	coated	
Chiralcel OX	coated	cellulose *tris*(4-chloro-3-methylphenylcarbamate)
Lux Cellulose-4	coated	
Chiralcel OJ (OJ-H)	coated	cellulose *tris*(4-methybenzoate)
Lux Cellulose-3	coated	
Chiralcel OK	coated	cellulose tricinnamate

[a)] Suppliers: Chiralcel and Chiralpak columns from Chiral Technologies (Daicel group); Lux columns from Phenomenex

[b)] coated on, or immobilized to silica gel

3.1 Planar chiral ferrocenes containing polar groups: additional materials

Table S3. HPLC enantioseparation of 2-acyl-1-dimethylaminomethylferrocenes **36a-q** with Chiralpak IE-3 and IC-3 under normal phase conditions [40]

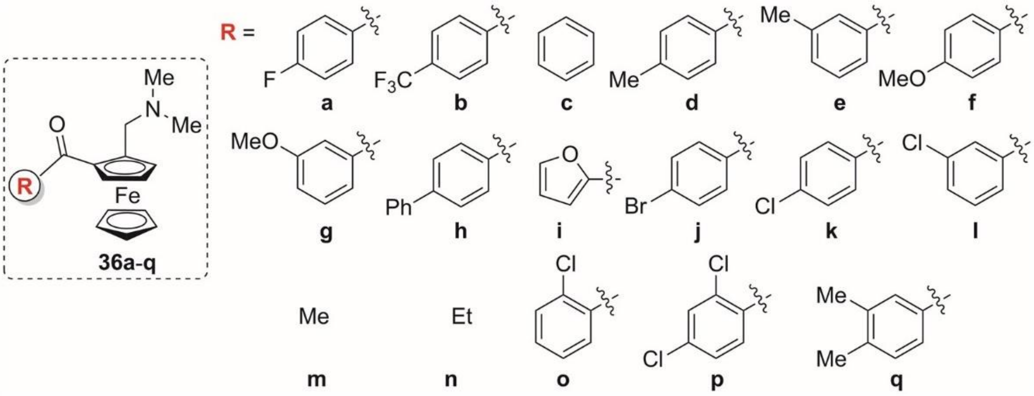

Fc	column	mobile phase, *FR* (ml/min) [a]	t_{R1} (min) [b]	t_{R2} (min) [b]	EEO [c]
**a**	IE	*n*-hexane/EtOH 8:2, 0.5	19.2	20.6	R_p-S_p
**b**	IE	*n*-hexane/EtOH 8:2, 0.5	11.0	11.5	R_p-S_p
**c**	IE	*n*-hexane/EtOH 7:3, 0.5	19.6	23.9	R_p-S_p
**d**	IE	*n*-hexane/EtOH 7:3, 0.5	20.1	21.8	R_p-S_p
**e**	IE	*n*-hexane/EtOH 6:4, 0.5	14.2	15.6	R_p-S_p
**f**	IE	*n*-hexane/EtOH 6:4, 0.5	19.4	21.8	R_p-S_p
**g**	IE	*n*-hexane/EtOH 6:4, 0.5	18.3	20.2	R_p-S_p
**h**	IE	*n*-hexane/EtOH 6:4, 0.5	21.3	25.8	R_p-S_p
**i**	IE	*n*-hexane/EtOH 6:4, 0.5	17.4	19.2	S_p-R_p
**j**	IE	*n*-hexane/EtOH 6:4, 0.5	15.5	19.1	R_p-S_p
**k**	IE	*n*-hexane/EtOH 6:4, 0.5	14.4	16.9	R_p-S_p
**l**	IE	*n*-hexane/EtOH 6:4, 0.5	12.7	14.1	R_p-S_p
**m**	IC	*n*-hexane/2-PrOH 7:3, 0.9	10.9	26.8	S_p-R_p
**n**	IC	*n*-hexane/2-PrOH 7:3, 0.9	8.3	14.6	S_p-R_p
**o**	IC	*n*-hexane/2-PrOH 7:3, 0.9	9.3	11.3	S_p-R_p
**p**	IC	*n*-hexane/2-PrOH 7:3, 0.9	8.8	10.7	S_p-R_p
**q**	IC	*n*-hexane/2-PrOH 7:3, 0.8	11.9	26.7	S_p-R_p

[a] flow rate

[b] retention time

[c] enantiomer elution order

Table S4. HPLC enantioseparation of planar chiral pyridine ferrocenopentadienone derivatives **70a**-**v** with Chiralcel OD-H, Chiralcel OJ-H, Chiralpak AD-H, and Chiralpak AS-H under normal phase conditions [29]

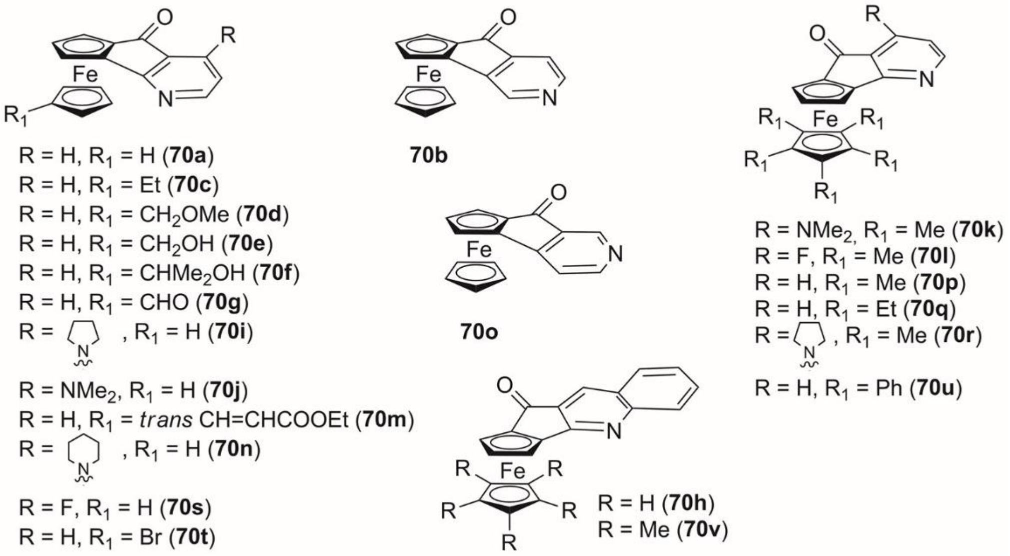

Fc	column	mobile phase, *FR* (ml/min) [a]	t_{R1} (min) [b]	t_{R2} (min) [b]	EEO [c]
**a**	OD-H	*n*-hexane/2-PrOH 90:10, 1.0	12.7	16.3	S_p-R_p
**b**	OD-H	*n*-hexane/2-PrOH 90:10, 1.0	14.1	15.6	R_p-S_p
**c**	OD-H	*n*-hexane/2-PrOH 90:10, 1.0	10.3	17.6	S_p-R_p
**d**	OD-H	*n*-hexane/2-PrOH 90:10, 1.0	18.1	24.5	S_p-R_p
**e**	OD-H	*n*-hexane/2-PrOH 90:10, 1.0	24.3	28.1	S_p-R_p
**f**	OD-H	*n*-hexane/2-PrOH 90:10, 1.0	12.0	14.0	S_p-R_p
**g**	OD-H	*n*-hexane/2-PrOH 90:10, 1.0	41.8	46.9	S_p-R_p
**h**	OD-H	*n*-hexane/2-PrOH 90:10, 1.0	14.8	16.7	R_p-S_p
**i**	OD-H	*n*-hexane/2-PrOH 90:10, 1.0	37.9	44.8	S_p-R_p
**j**	OD-H	*n*-hexane/2-PrOH 90:10, 1.0	22.2	26.0	S_p-R_p
**k**	OD-H	*n*-hexane/2-PrOH 90:10, 1.0	10.9	21.2	S_p-R_p
**l**	OD-H	*n*-hexane/2-PrOH 90:10, 1.0	7.7	11.0	S_p-R_p
**m**	OJ-H	*n*-hexane/2-PrOH 95:5, 1.0	34.1	40.5	R_p-S_p
**n**	AD-H	*n*-hexane/2-PrOH 90:10, 1.0	8.9	9.8	S_p-R_p
**o**	AD-H	*n*-hexane/2-PrOH 90:10, 1.0	14.4	17.4	R_p-S_p
**p**	AD-H	*n*-hexane/2-PrOH 90:10, 1.0	5.5	5.8	S_p-R_p
**q**	AD-H	*n*-hexane/2-PrOH 90:10, 1.0	5.3	6.4	S_p-R_p
**r**	AD-H	*n*-hexane/2-PrOH 90:10, 1.0	8.6	9.9	R_p-S_p
**s**	AD-H	*n*-hexane/2-PrOH 98:2, 1.0	14.2	15.1	S_p-R_p
**t**	AD-H	*n*-hexane/2-PrOH 98:2, 0.4	58.5	64.3	S_p-R_p
**u**	AD-H	*n*-hexane/2-PrOH 98:2, 0.4	23.5	25.3	S_p-R_p
**v**	AS-H	*n*-hexane/2-PrOH 98:2, 0.4	25.9	33.1	R_p-S_p

[a] flow rate

[b] retention time

[c] enantiomer elution order

Table S5. Enantioseparation of *N*-cumyl-N-ethylferrocenecarboxamide **84**, of *N*-ethylferrocenecarboxamides **85a**-**f**, of methoxycarbonylferrocenes **86a**-**c**, of ferrocenylpiperidinone **87**, of ferrocenyldihydroazepinone **88** and ferrocenyltetrahydroazepinone **89** by using the Chiralcel OD, as chiral column, and *n*-hexane/2-PrOH mixtures as mobile phases [184]

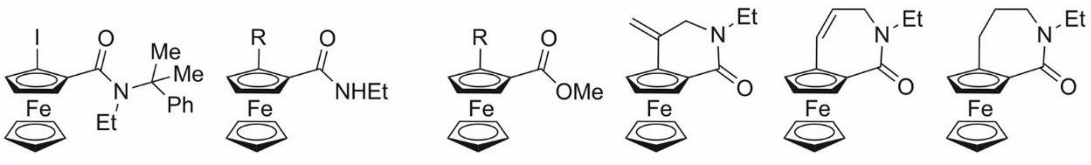

84

R = Me (**85a**)
R = SMe (**85b**)
R = I (**85c**)
R = $SiMe_3$ (**85d**)
R = $CH_2OCH_2CF_3$ (**85e**)
R = CH_2OCH_3 (**85f**)

R = Me (**86a**)
R = $SiMe_3$ (**86b**)
R = I (**86c**)

87

88

89

**Fc**	**mobile phase, *FR* (ml/min)** [a)]	t_{R1} **(min)** [b)]	t_{R2} **(min)** [b)]	**EEO** [c)]
**84**	*n*-hexane/2-PrOH 99:1, 0.4	31.1	32.8	R_p-S_p
**85a**	*n*-hexane/2-PrOH 90:10, 1.0	9.6	20.9	S_p-R_p
**85b**	*n*-hexane/2-PrOH 90:10, 1.0	6.8	10.3	R_p-S_p
**85c**	*n*-hexane/2-PrOH 85:15, 1.0	13.2	15.7	R_p-S_p
**85d**	*n*-hexane/2-PrOH 90:10, 1.0	6.4	13.0	R_p-S_p
**85e**	*n*-hexane/2-PrOH 90:10, 1.0	10.3	28.9	S_p-R_p
**85f**	*n*-hexane/2-PrOH 90:10, 1.0	10.9	19.5	S_p-R_p
**86a**	*n*-hexane/2-PrOH 96:4, 1.0	6.2	7.2	S_p-R_p
**86b**	*n*-hexane/2-PrOH 99:1, 0.35	14.1	16.4	R_p-S_p
**86c**	*n*-hexane/2-PrOH 90:10, 0.8	7.1	9.1	R_p-S_p
**87**	*n*-hexane/2-PrOH 90:10, 1.0	10.5	12.0	R_p-S_p
**88**	*n*-hexane/2-PrOH 90:10, 1.0	8.6	13.2	R_p-S_p
**89**	*n*-hexane/2-PrOH 98:2, 0.4	9.5	11.8	R_p-S_p

[a)] flow rate

[b)] retention time

[c)] enantiomer elution order

Table S6. HPLC enantioseparation of planar chiral alkylated ferrocene carboxamides **37a**-**h** and **91**-**97** with Chiralpak IA, Chiralpak AD, Chiralpak IG, Chiralcel OD, and Chiralpak IB under normal phase conditions [146]

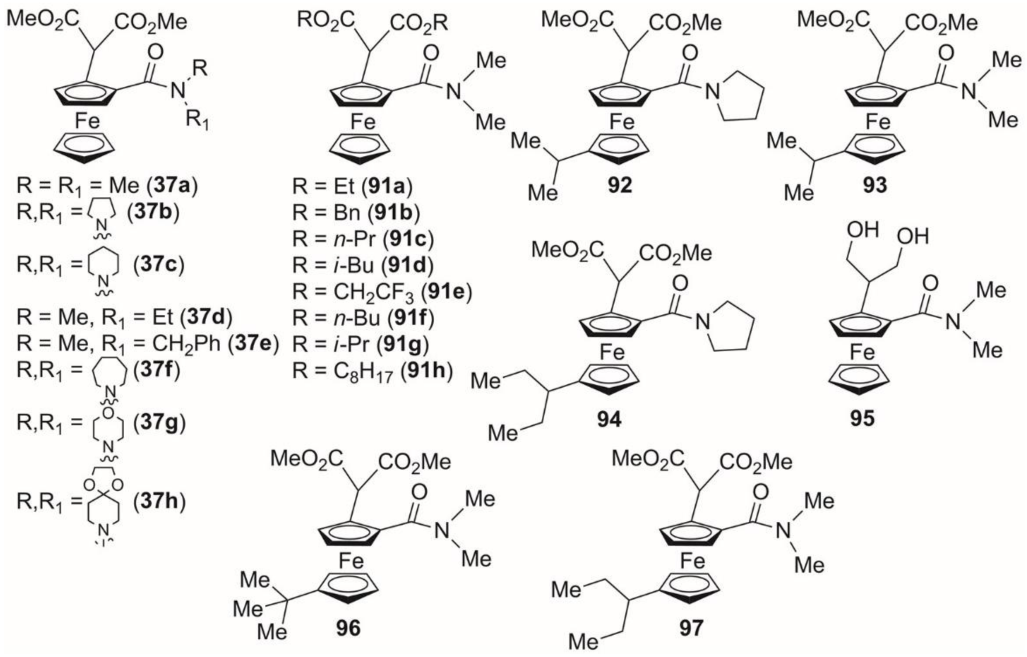

Fc	column	mobile phase, *FR* (ml/min) a)	t_{R1} (min) b)	t_{R2} (min) b)	EEO c)
**37a**	IA	*n*-hexane/2-PrOH 90:10, 1.0	11.9	13.1	R_p-S_p
**37b**	IA	*n*-hexane/2-PrOH 95:5, 1.0	25.7	34.0	R_p-S_p
**37c**	IA	*n*-hexane/2-PrOH 95:5, 1.0	16.9	20.0	R_p-S_p
**37d**	AD	*n*-hexane/2-PrOH 95:5, 1.0	18.1	22.7	R_p-S_p
**37e**	AD	*n*-hexane/2-PrOH 95:5, 1.0	32.0	34.9	R_p-S_p
**37f**	OD	*n*-hexane/2-PrOH 95:5, 1.0	17.4	25.7	S_p-R_p
**37g**	IB	*n*-hexane/2-PrOH 95:5, 1.0	22.9	24.6	R_p-S_p
**37h**	IB	*n*-hexane/2-PrOH 95:5, 1.0	18.1	22.6	S_p-R_p
**91a**	IA	*n*-hexane/2-PrOH 95:5, 1.0	16.6	18.1	R_p-S_p
**91b**	IA	*n*-hexane/2-PrOH 95:5, 1.0	42.7	49.6	S_p-R_p
**91c**	IA	*n*-hexane/2-PrOH 98:2, 1.0	23.0	26.9	R_p-S_p
**91d**	IA	*n*-hexane/2-PrOH 98:2, 1.0	17.8	21.6	R_p-S_p
**91e**	AD	*n*-hexane/2-PrOH 95:5, 1.0	14.1	17.2	R_p-S_p
**91f**	AD	*n*-hexane/2-PrOH 98:2, 1.0	12.8	21.9	R_p-S_p
**91g**	OD	*n*-hexane/2-PrOH 95:5, 1.0	8.0	16.4	S_p-R_p
**91h**	OD	*n*-hexane/2-PrOH 95:5, 1.0	7.6	28.5	S_p-R_p
**92**	IA	*n*-hexane/2-PrOH 95:5, 1.0	19.5	22.4	R_p-S_p
**93**	AD	*n*-hexane/2-PrOH 95:5, 1.0	18.5	20.0	R_p-S_p
**94**	IG	*n*-hexane/2-PrOH 95:5, 1.0	16.6	18.2	R_p-S_p
**95**	OD	*n*-hexane/2-PrOH 90:10, 1.0	9.8	11.7	S_p-R_p
**96**	OD	*n*-hexane/2-PrOH 95:5, 1.0	11.9	16.8	S_p-R_p
**97**	IB	*n*-hexane/2-PrOH 95:5, 1.0	9.1	18.0	S_p-R_p

a) flow rate

b) retention time

c) enantiomer elution order

Table S7. HPLC enantioseparation of planar chiral borylated ferrocenes **98a**-**y**, **99a-f** and **100**-**102**, and ferrocene carboxamide **103** with Chiralpak AD-H, Chiralpak AD, Chiralpak IE, Chiralpak IF, Chiralpak IC under normal phase conditions [42]

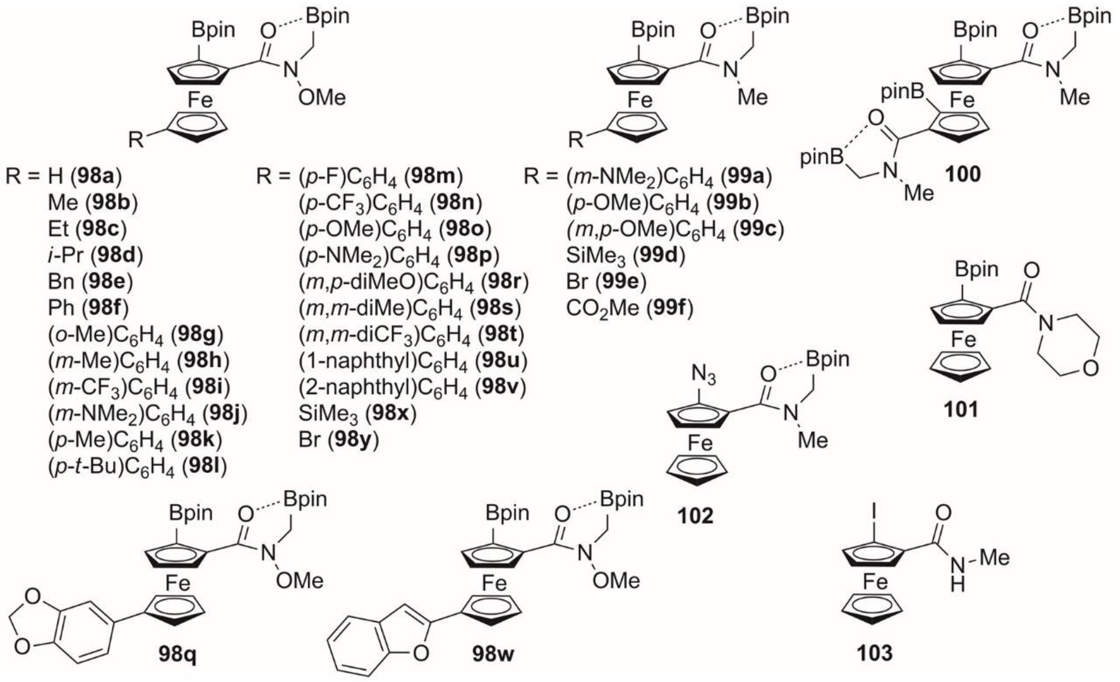

Fc	column	mobile phase, *FR* (ml/min) a)	t_{R1} (min) b)	t_{R2} (min) b)
**98a**	IC	*n*-hexane/2-PrOH 90:10, 0.5	13.2	15.1
**98b**	IE	*n*-hexane/2-PrOH 95:5, 1.0	21.3	23.6
**98c**	IE	*n*-hexane/2-PrOH 95:5, 0.5	36.6	41.7
**98d**	IC	*n*-hexane/2-PrOH 95:5, 0.5	13.6	14.6
**98e**	IC	*n*-hexane/2-PrOH 90:10, 0.5	12.9	14.3
**98f**	IC	*n*-hexane/2-PrOH 95:5, 0.5	23.5	26.6
**98g**	IC	*n*-hexane/2-PrOH 95:5, 0.5	25.0	28.8
**98h**	IC	*n*-hexane/2-PrOH 95:5, 0.5	24.7	27.5
**98i**	IC	*n*-hexane/2-PrOH 90:10, 0.5	15.1	16.3
**98j**	AD-H	*n*-hexane/2-PrOH 95:5, 0.5	14.1	20.1
**98k**	IC	*n*-hexane/2-PrOH 95:5, 0.5	24.4	27.4
**98l**	IC	*n*-hexane/2-PrOH 90:10, 0.5	12.1	14.3
**98m**	IC	*n*-hexane/2-PrOH 95:5, 0.5	19.3	21.8
**98n**	IC	*n*-hexane/2-PrOH 95:5, 0.5	14.2	15.2
**98o**	IE	*n*-hexane/2-PrOH 90:10, 1.0	15.6	19.6
**98p**	IC	*n*-hexane/2-PrOH 90:10, 0.5	24.9	31.6
**98q**	IC	*n*-hexane/2-PrOH 90:10, 0.5	22.0	24.6
**98r**	IC	*n*-hexane/2-PrOH 90:10, 1.0	28.9	35.6
**98s**	IF	*n*-hexane/2-PrOH 90:10, 0.5	19.7	23.0
**98t**	IF	*n*-hexane/2-PrOH 98:2, 0.5	23.5	27.5
**98u**	IC	*n*-hexane/2-PrOH 90:10, 0.5	14.7	16.1
**98v**	IC	*n*-hexane/2-PrOH 90:10, 0.5	15.0	16.6
**98w**	IC	*n*-hexane/2-PrOH 95:5, 0.5	20.0	22.1
**98x**	IF	*n*-hexane/2-PrOH 98:2, 0.5	28.6	34.7
**98y**	IC	*n*-hexane/2-PrOH 90:10, 0.5	12.0	12.7

a) flow rate

b) retention time

Table S7. *continued*

Fc	column	mobile phase, *FR* (ml/min) [a]	t_{R1} (min) [b]	t_{R2} (min) [b]
**99a**	IE	*n*-hexane/2-PrOH 90:10, 1.0	15.8	18.5
**99b**	IE	*n*-hexane/2-PrOH 90:10, 0.5	28.2	31.4
**99c**	IE	*n*-hexane/2-PrOH 90:10, 1.0	34.4	43.9
**99d**	IC	*n*-hexane/2-PrOH 90:10, 0.5	12.2	16.2
**99e**	IC	*n*-hexane/2-PrOH 90:10, 0.5	15.1	20.6
**99f**	IC	*n*-hexane/2-PrOH 90:10, 0.5	13.3	16.4
**100**	IC	*n*-hexane/2-PrOH 90:10, 1.0	21.7	40.4
**101**	IC	*n*-hexane/2-PrOH 95:5, 0.5	9.7	12.4
**102**	AD	*n*-hexane/2-PrOH 95:5, 0.5	20.8	22.6
**103**	AD	*n*-hexane/2-PrOH 90:10, 1.0	8.7	9.4

[a] flow rate

[b] retention time

Table S8. HPLC enantioseparation of planar chiral ferrocene-fused pyridones **33a**-**u**, **104a-k** and **105** with Chiralpak AD-H, Chiralpak IG, Chiralcel OD-H, Chiralpak IC under normal phase conditions [142]

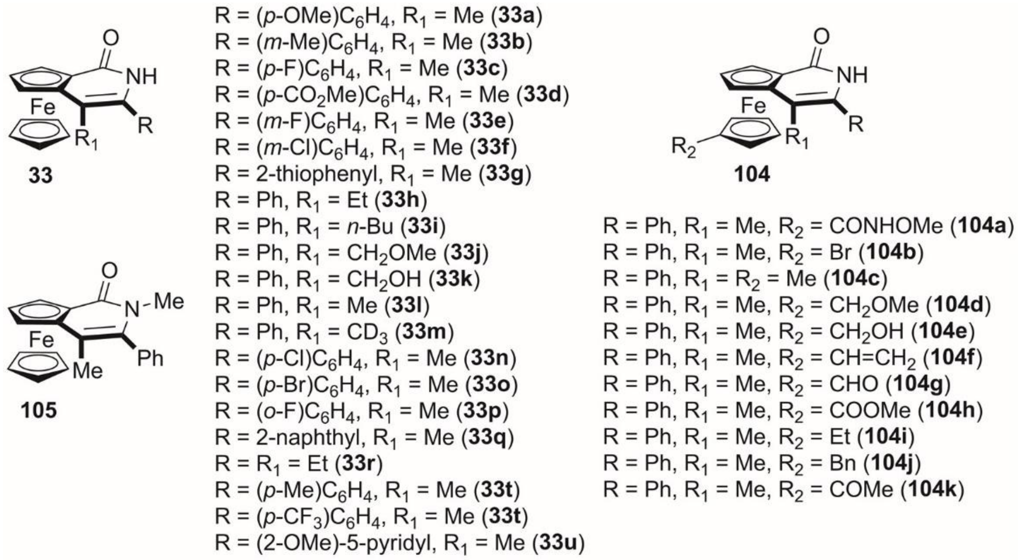

Fc	column	mobile phase, *FR* (ml/min) a)	t_{R1} (min) b)	t_{R2} (min) b)
**33a**	AD-H	*n*-hexane/2-PrOH 85:15, 1.0	10.9	12.8
**33b**	AD-H	*n*-hexane/2-PrOH 85:15, 1.0	6.4	8.7
**33c**	AD-H	*n*-hexane/2-PrOH 90:10, 1.0	11.5	13.2
**33d**	AD-H	*n*-hexane/2-PrOH 90:10, 1.0	19.9	24.8
**33e**	AD-H	*n*-hexane/2-PrOH 90:10, 1.0	8.4	12.1
**33f**	AD-H	*n*-hexane/2-PrOH 90:10, 1.0	8.6	13.7
**33g**	AD-H	*n*-hexane/2-PrOH 90:10, 1.0	12.5	13.8
**33h**	AD-H	*n*-hexane/2-PrOH 90:10, 1.0	9.1	14.3
**33i**	AD-H	*n*-hexane/2-PrOH 90:10, 1.0	6.8	8.6
**33j**	AD-H	*n*-hexane/2-PrOH 90:10, 1.0	12.4	14.3
**33k**	AD-H	*n*-hexane/2-PrOH 90:10, 1.0	24.7	30.2
**33l**	AD-H	*n*-hexane/2-PrOH 95:5, 1.0	18.5	23.4
**33m**	AD-H	*n*-hexane/2-PrOH 95:5, 1.0	18.5	23.3
**33n**	AD-H	*n*-hexane/2-PrOH 95:5, 1.0	21.2	23.5
**33o**	AD-H	*n*-hexane/2-PrOH 95:5, 1.0	22.4	25.3
**33p**	AD-H	*n*-hexane/2-PrOH 95:5, 1.0	21.1	23.1
**33q**	AD-H	*n*-hexane/2-PrOH 95:5, 1.0	30.7	37.87
**33r**	AD-H	*n*-hexane/2-PrOH 95:5, 0.7	15.6	17.4
**33s**	AD-H	*n*-hexane/2-PrOH 97:3, 0.7	51.3	57.2
**33t**	IC	*n*-hexane/2-PrOH 90:10, 1.0	27.0	30.0
**33u**	IG	*n*-hexane/2-PrOH 80:20, 1.0	19.1	24.1
**104a**	AD-H	*n*-hexane/2-PrOH 80:20, 1.0	6.4	10.4
**104b**	AD-H	*n*-hexane/2-PrOH 90:10, 1.0	11.4	12.8
**104c**	AD-H	*n*-hexane/2-PrOH 90:10, 1.0	8.8	11.3
**104d**	AD-H	*n*-hexane/2-PrOH 90:10, 1.0	12.5	14.5
**104e**	AD-H	*n*-hexane/2-PrOH 90:10, 1.0	18.9	22.4
**104f**	AD-H	*n*-hexane/2-PrOH 90:10, 1.0	9.1	11.1
**104g**	AD-H	*n*-hexane/2-PrOH 90:10, 1.0	25.1	28.9
**104h**	AD-H	*n*-hexane/2-PrOH 90:10, 1.0	16.9	24.2
**104i**	OD-H	*n*-hexane/2-PrOH 90:10, 1.0	7.4	10.5

a) flow rate

b) retention time

Table S8. *continued*

Fc	column	mobile phase, *FR* (ml/min) a)	t_{R1} (min) b)	t_{R2} (min) b)
**104j**	OD-H	*n*-hexane/2-PrOH 85:15, 1.0	7.2	10.2
**104k**	OD-H	*n*-hexane/2-PrOH 80:20, 1.0	8.3	9.5
**105**	IG	*n*-hexane/2-PrOH 85:15, 1.0	5.8	6.9

a) flow rate

b) retention time

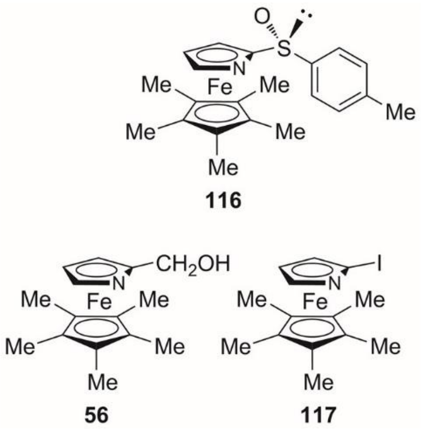

Figure S8. Structures of azaferrocenes **56**, **116** and **117**.

3.2 Planar chiral ferrocenes containing aromatic groups and extended π-clouds: additional materials

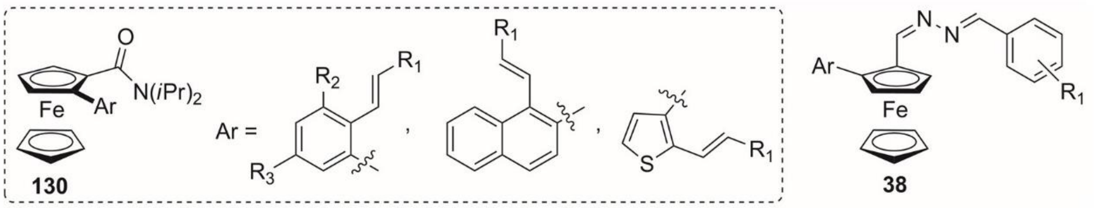

Figure S9. Structures of the planar chiral ferrocene series **130** and **38**.
